# FlashDeconv enables atlas-scale, multi-resolution spatial deconvolution via structure-preserving sketching

**DOI:** 10.64898/2025.12.22.696108

**Published:** 2025-12-25

**Authors:** Chen Yang, Xianyang Zhang, Jun Chen

**Affiliations:** 1 Department of Statistics, Texas A&M University, College Station, Texas, 77843, USA.; 2 Division of Computational Biology, Department of Quantitative Health Sciences, Mayo Clinic, Rochester, Minnesota, 55905, USA.

**Keywords:** Spatial transcriptomics, Cell type deconvolution, Visium HD, Atlas-scale analysis, Randomized sketching

## Abstract

Atlas-scale spatial transcriptomics requires deconvolution methods that preserve rare biological signals without prohibitive computational costs. Here, we introduce FlashDeconv, a framework built on structure-preserving randomized sketching. Unlike variance-based methods that conflate biological information with population abundance, FlashDeconv employs leverage-score importance sampling to prioritize transcriptomically distinct markers—preserving rare cell type signals that standard feature selection discards. Benchmarking demonstrates accuracy comparable to top-tier Bayesian methods while accelerating inference by orders of magnitude. Applied to human ovarian cancer cohorts, FlashDeconv reproduces clinical response signatures in seconds, enabling rapid patient stratification. This throughput also enables systematic scale-space exploration: we define a “resolution horizon” (8–16 ***μ***m) beyond which cellular co-localization signals undergo sign inversion due to geometric mixing. Operating below this horizon, FlashDeconv uncovers cryptic Tuft cell niches enriched for intestinal stem cells—biological architecture obscured by both variance-based feature selection and coarse spatial binning. FlashDeconv provides a scalable, mathematically grounded framework for atlas-scale spatial discovery.

## Introduction

Spatial transcriptomics (ST) technologies, such as Stereo-seq [1], Xenium [2], and Visium HD [3], are transforming our understanding of tissue architecture by mapping gene expression at subcellular resolution across increasingly large fields of view. As these technologies scale to atlas-level datasets comprising millions of spots or cells, the computational burden of analyzing such data has become a critical bottleneck. A fundamental task in ST analysis is cell type deconvolution, which infers the proportional composition of cell types within each spatial measurement unit by leveraging single-cell RNA sequencing (scRNA-seq) references.

Existing deconvolution methods can be broadly distinguished by their modeling paradigms: generative probabilistic modeling versus constrained regression. Generative methods such as Cell2Location [4] and stereoscope [5] achieve high accuracy by explicitly modeling count data distributions (e.g., Negative Binomial) via variational inference or maximum a posteriori (MAP) estimation, providing rigorous uncertainty quantification that is particularly valuable for small-scale studies requiring careful statistical inference. However, these methods require extensive iterative training, with runtimes scaling from hours to days for million-scale datasets. In contrast, regression-based approaches formulate the problem as constrained minimization, offering faster inference. This category includes the baseline NNLS, weighted variants such as MuSiC [6] and SpatialDWLS [7], likelihood-based regression (RCTD [8]), NMF decomposition (SPOTlight [9]), and deep-learning alignment (Tangram [10]). Benchmarks indicate that many methods fail to outperform NNLS consistently; only Cell2Location, RCTD, and MuSiC exceed baseline performance across all evaluation metrics [11]. However, most regression approaches treat spatial spots as independent observations, ignoring local tissue continuity. Methods that do incorporate spatial structure, such as CARD [12] with its conditional autoregressive (CAR) prior, typically rely on dense covariance matrices ($O\left(N^{2}\right)$ memory), precluding analysis of emerging high-resolution platforms with millions of spots.

Here, we present FlashDeconv, a deconvolution framework that achieves accuracy, spatial awareness, and linear scalability simultaneously. FlashDeconv leverages Randomized Numerical Linear Algebra (RandNLA) to compress the high-dimensional gene expression space (G≈20,000) into a compact “sketch” (d≈512) using a structure-preserving sketching technique. Unlike standard random projections that risk losing signals from rare cell types, our approach utilizes leverage-score importance sampling to effectively preserve biological heterogeneity. Here, “structure” refers to the geometric organization of cell types in gene expression space, which leverage scores quantify by measuring each gene’s contribution to distinguishing cell type signatures. Combined with a Log-CPM data representation and a sparse graph Laplacian regularizer that scales as O(N) rather than $O\left(N^{2}\right)$, FlashDeconv achieves accuracy comparable to top-tier Bayesian methods on synthetic benchmarks while reducing runtime by orders of magnitude, enabling atlas-scale analysis on standard commodity hardware.

A critical challenge in deconvolution is that traditional dimension reduction methods—such as Principal Component Analysis (PCA) or Highly Variable Genes (HVG) selection—rely on *variance* as a proxy for biological information [13, 14]. Intuitively, variance measures how “loud” a gene is across the dataset—a quantity naturally dominated by abundant cell types. In contrast, leverage scores measure *distinctiveness*: whether a gene defines a unique direction in the transcriptomic space, regardless of how many cells express it. However, gene variance is intrinsically confounded with expression magnitude and cell type abundance: highly abundant cell types (e.g., 30% of cells) naturally dominate the variance spectrum, inherently underweighting signals from rare but biologically critical populations. Such rare populations—including cancer stem cells comprising <1% of tumors [15, 16] and vascular endothelial cells that orchestrate tissue-specific stem cell niches [17]—often determine tissue fate and disease progression, yet their markers are systematically underrepresented by variance-based feature selection [18, 19]. This limitation has motivated alternative approaches, including Gini-index-based gene selection for rare cell detection [20] and deviance-based feature ranking that avoids the false variability introduced by HVG selection [21]. To overcome this limitation, FlashDeconv employs *leverage scores*, a geometric measure of gene importance derived from the singular value decomposition (SVD) of the reference matrix. Unlike variance, leverage scores quantify how much each gene contributes to the discriminative *structure* among cell types, *independently* of expression level or population frequency. In essence, leverage captures *transcriptomic distinctiveness*—whether a cell type’s markers define a unique direction in gene expression space—rather than simply reflecting how many cells of that type exist. We validate this principle through a series of experiments that form a comprehensive evidence chain. First, an “abundance invariance” stress test demonstrates that when dominant cell types are artificially downsampled from 27% to 0.4% of the population, variance-based marker ranking degrades by over 50%, while leverage-score ranking remains stable—proving true mathematical decoupling of biological identity from population frequency. Second, by mapping genes into a variance-leverage plane, we identify structurally informative genes (low variance, high leverage; hereafter “GOLD”) representing markers of rare anatomical structures that are systematically underweighted by standard HVG selection. Functional enrichment analysis reveals these GOLD genes are significantly enriched for vascular development pathways (*angiogenesis*, FDR-adjusted $p=2.8\times 10^{-6}$), while variance-dominated genes (high variance, low leverage; hereafter “NOISE”) show no functional enrichment and contain 6-fold more unannotated transcripts. Third, spatial visualization confirms that GOLD genes reconstruct clear anatomical structures on tissue sections, whereas NOISE genes exhibit random speckle-like patterns. Finally, systematic ablation experiments across 54 benchmark datasets demonstrate that leverage-weighted sketching improves rare cell type detection by 124–197% compared to PCA, uniform sketching, and HVG selection, confirming that these theoretical insights translate directly to downstream performance. Together, these results establish that leverage scores provide a principled, abundance-independent measure of biological informativeness, forming the mathematical foundation for FlashDeconv’s ability to preserve critical signals during dimension reduction.

## Results

### The FlashDeconv framework

**Fig. 1:**
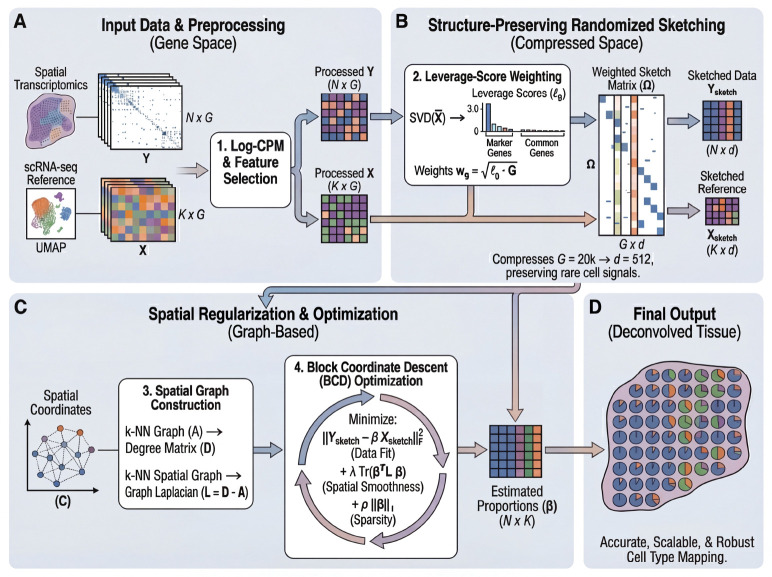
Overview of the FlashDeconv framework. **(a)** Input data and preprocessing. Spatial transcriptomics data Y(N×G) and scRNA-seq reference signatures X(K×G) are normalized using Log-CPM transformation. Gene selection combines HVGs from Y with cell-type markers from X (whole transcriptome), yielding the union set 𝒢. **(b)** Structure-preserving sketching. Leverage scores $\ell_{g}$ are computed from the SVD of the reference matrix to identify discriminative genes. A weighted sketch matrix Ω(G×d) compresses the gene space from G≈20,000 to d=512 dimensions while preserving rare cell type signals. **(c)** Spatial regularization and optimization. A k-nearest neighbor graph is constructed from spatial coordinates, and the graph Laplacian L enforces spatial smoothness. The optimization problem minimizes reconstruction error with spatial and sparsity regularization via block coordinate descent (BCD). **(d)** Final output. FlashDeconv produces cell type proportion estimates β(N×K) for each spatial location, enabling accurate, scalable, and robust cell type mapping across the tissue.

FlashDeconv formulates spatial deconvolution as a constrained optimization problem in a compressed feature space (Fig. 1). The framework consists of three key design choices:

First, to address the extreme sparsity and mean-variance dependency of ST data, we employ a Log-CPM (Counts Per Million) transformation. While Pearson residuals are statistically principled for negative binomial count data [22], Log-CPM offers specific advantages for $L_{2}$-based sketching: its bounded norm prevents high-expression genes from dominating the sketch space, while its logarithmic compression stabilizes the extreme dynamic range of spatial count data. This represents an engineering trade-off—sacrificing some statistical optimality for compatibility with randomized compression (Supplementary Note 2).

**Fig. 2:**
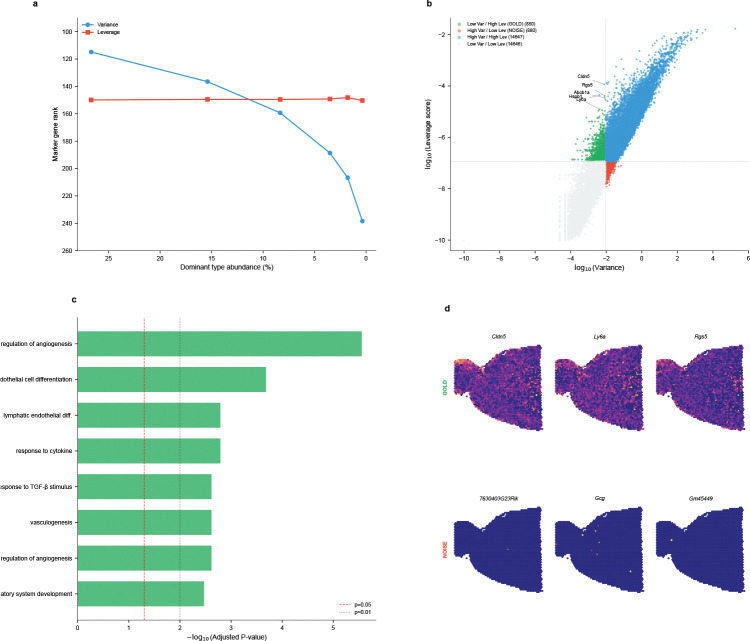
Leverage scores decouple biological identity from population abundance. **(a)** Abundance invariance test. Ranking stability of oligodendrocyte markers (top 20 genes by expression in oligodendrocytes) in mouse brain scRNA-seq (31,053 genes) as cell population is downsampled from 26.7% to 0.4%. Rank denotes average position when all genes are sorted by score (rank 1 = highest). Variance-based ranking (blue) degrades from rank 115 to 240 as abundance decreases—a two-fold deterioration. Leverage-score ranking (red) remains stable at rank ~150 regardless of population size, demonstrating true decoupling of biological identity from numerical prevalence. **(b)** The variance-leverage plane. Classification of 31,053 genes by variance (x-axis) and leverage score (y-axis). Four quadrants emerge: structurally informative “GOLD” genes (green, low variance/high leverage) include vascular markers (*Cldn5*, *Rgs5*, *Ly6a*, *Abcb1a*, *Hspb1*) that define rare anatomical structures; variance-dominated “NOISE” genes (red, high variance/low leverage) contain 35% unannotated *Gm*-series transcripts compared to only 6% in the GOLD set, indicating that high variance alone does not ensure cell-type discriminative power. **(c)** Functional enrichment analysis. GO Biological Process enrichment reveals GOLD genes are significantly enriched for regulation of angiogenesis (FDR-adjusted $p=2.8\times 10^{-6}$), endothelial cell differentiation (FDR-adjusted $p=2.1\times 10^{-4}$), vasculogenesis, and blood vessel morphogenesis. NOISE genes show zero significant GO terms at FDR-adjusted p<0.05. Genome-wide cell type specificity analysis further confirms that GOLD genes systematically target rare populations (median 0.27% abundance) versus NOISE genes (0.51%; $p=3.25\times 10^{-25}$), with Endothelial cells as the top target—validating leverage as an unsupervised metric for biological distinctiveness. **(d)** Spatial verification on Visium. Top row: GOLD genes (*Cldn5*, *Ly6a*, *Rgs5*) reconstruct clear vascular anatomical structures on mouse brain Visium sections (spatial structure score = 1.33). Bottom row: NOISE genes exhibit random, speckle-like distribution patterns (structure score = 0.87; Mann-Whitney $p=5.6\times 10^{-5}$). This visual contrast demonstrates that leverage selects for genuine biological structure rather than technical variation.

Second, we tackle the computational redundancy of gene expression via structure-preserving randomized sketching. Instead of solving the regression on all G genes, we project the data into a lower-dimensional subspace using a sparse CountSketch matrix Ω. Unlike PCA, which maximizes explained variance and may obscure cell types that contribute little to global variance, our sketching matrix satisfies the Johnson-Lindenstrauss property [23], guaranteeing that Euclidean distances between cell type signatures are maintained in the compressed space with high probability. Crucially, the projection is weighted by statistical leverage scores [24, 25] derived from the single-cell reference. This ensures that marker genes defining transcriptomically distinct cell types—which often have high leverage despite low variance—are preserved with high probability, avoiding the signal loss that can occur with uniform subsampling or variance-based feature selection. For example, in liver scRNA-seq data, Central Vein Endothelial cells constitute only 2% of cells, yet their marker gene *Rspo3* achieves the highest leverage score (0.0105) among all genes due to its unique expression pattern that sharply distinguishes this population from all others. In contrast, Hepatocyte markers like *Egfr* exhibit low leverage scores (0.0007) despite high specificity, because Hepatocytes share metabolic gene programs with Cholangiocytes, reducing their geometric distinctiveness in the reference space. This illustrates that leverage captures *functional distinctiveness* rather than abundance: Hepatocytes are abundant but transcriptionally overlap with other lineages, while Central Vein Endothelial cells are rare but transcriptomically unique. Standard variance-based methods (PCA, HVG) show no statistical difference between functionally distinct and overlapping markers (Mann-Whitney U test, p=0.97), whereas leverage-score ranking successfully identifies the structurally informative genes (Mann-Whitney U test, p=0.0062). We provide comprehensive validation of this principle in Section 2, demonstrating through abundance invariance tests, functional enrichment analysis, and spatial visualization that leverage scores consistently identify biologically meaningful genes across tissues. Critically, leverage-based weighting also addresses the fundamental challenge of hash collisions in randomized sketching. In standard random projections, high-abundance “housekeeping” genes contribute disproportionately to the total squared $L_{2}$ magnitude, dominating the sketch space. When such a gene randomly hashes (collides) into the same sketch dimension as a rare cell marker, the massive collision noise mathematically overwhelms the rare signal. Leverage-based importance weighting acts as a *selective amplifier*: genes are scaled by $\sqrt{\ell_{g}\cdot G}$ before entering the sketch, where $\ell_{g}$ is the leverage score. Crucially, high-leverage genes (often markers of rare cell types) receive proportionally stronger amplification *relative to their baseline magnitude* than low-leverage genes. Empirically, we observe that low-variance/high-leverage “GOLD” genes exhibit 3–12× higher leverage-to-magnitude ratios than high-variance/low-leverage “NOISE” genes across six tissues (mean 6.2×; Fig. 2B), yielding asymmetric signal-to-interference ratio (SIR) gains during sketching. This asymmetry—not global magnitude equalization—ensures that when hash collisions occur (i.e., when multiple genes are randomly assigned to the same sketch dimension and their contributions are summed), rare marker signals are preferentially preserved while technical noise is relatively attenuated. Mathematically, this selective weighting mitigates hash collision variance [26] by ensuring that marker genes maintain adequate signal-to-noise ratio in the compressed space.

Third, we incorporate spatial information using a graph Laplacian regularizer. Unlike CARD, which constructs and operates on a dense N×N spatial kernel matrix with $O\left(N^{2}\right)$ memory and $O\left(N^{2}\cdot K\right)$ per-iteration complexity (where K is the number of cell types), our approach imposes spatial smoothness via a sparse penalty term $Tr\left(\beta^{T}L\beta \right)$—a formulation originally developed for structured genomic data [27] and shared with recent spatial-aware methods such as SDePER [28] and FAST [29]—where L is the graph Laplacian of the spatial neighbor network. This regularization induces local smoothness that is mathematically equivalent to a Gaussian Markov Random Field (GMRF) [30] but requires only sparse matrix operations. Because each spot connects to only k neighbors (typically k≈10), the spatial term scales as O(N⋅k) rather than $O\left(N^{2}\right)$, yielding linear time complexity. This formulation allows FlashDeconv to model spatial autocorrelation across millions of spots using a fast Block Coordinate Descent (BCD) algorithm, a scale where dense matrix inversions are computationally prohibitive. Biologically, this regularization encodes the intuition that tissue composition varies smoothly: for a spot with sparse sequencing depth, the algorithm borrows strength from its spatial neighbors, allowing coherent tissue structures to emerge from noisy measurements.

### Leverage decouples biological identity from population abundance

A fundamental premise of FlashDeconv is that statistical variance conflates biological signal with population frequency, systematically disadvantaging rare cell types. To systematically test this claim, we designed a series of experiments that trace the evidence from mathematical principle through molecular function to spatial phenotype.

#### Abundance invariance test.

We performed an *in silico* stress test using the mouse brain scRNA-seq reference (40,532 cells, 59 cell types, 31,053 genes). Starting from the native cell type distribution, we artificially downsampled the dominant oligodendrocyte population from 26.7% to 0.4%—a 67-fold reduction—while keeping all other populations constant. At each downsampling level, we identified the top 20 marker genes (by expression level in oligodendrocytes), computed both variance-based (normalized dispersion [31]) and leverage-based rankings across all genes, and tracked how the average marker rank changed (Fig. 2A). The results reveal a striking divergence: variance-based ranking degraded linearly with abundance, with marker rank dropping from 115 to 240 as the population shrank—a two-fold deterioration. In contrast, leverage-score ranking remained stable at rank ~150 throughout the entire range, demonstrating that leverage successfully decouples a gene’s discriminative power from its parent cell type’s numerical prevalence. Quantitatively, the stability can be attributed to leverage’s reduced coupling to mean expression: while gene variance correlates almost perfectly with mean expression (Spearman ρ=0.998), leverage scores show substantially greater spread around this relationship (ρ=0.965), enabling identification of low-abundance but high-specificity markers (Supplementary Fig. S5).

#### Gene quadrant analysis reveals systematic bias.

To understand the broader implications of this decoupling, we mapped all 31,053 genes into a variance-leverage coordinate system (Fig. 2B). Four distinct quadrants emerged, defined by median log-transformed values to ensure equal partitioning without arbitrary threshold selection. The “GOLD” quadrant (low variance, high leverage) contains genes that standard HVG selection would discard but that carry high discriminative power—including classic vascular markers such as *Cldn5* (claudin-5, tight junction protein), *Rgs5* (pericyte marker), *Ly6a* (stem cell antigen), *Abcb1a* (blood-brain barrier transporter), and *Hspb1* (heat shock protein). These genes define rare but anatomically critical cell populations: brain endothelial cells and pericytes constitute <3% of brain tissue yet form the blood-brain barrier essential for neural function. Conversely, the “NOISE” quadrant (high variance, low leverage) contains genes with high variance but low cell-type specificity: 35% are unannotated *Gm*-series transcripts (predicted genes with unknown function) compared to only 6% in the GOLD set—a 6-fold enrichment indicating that variance-based selection systematically prioritizes genes lacking discriminative power for cell type identification.

#### Functional enrichment provides molecular validation.

To confirm that the GOLD/NOISE distinction reflects genuine biological differences rather than statistical coincidence, we performed Gene Ontology enrichment analysis using Enrichr [32, 33] with Benjamini-Hochberg FDR correction on both gene sets (Fig. 2C). GOLD genes showed highly significant enrichment for vascular biology: *regulation of angiogenesis* (FDR-adjusted $p=2.8\times 10^{-6}$), *endothelial cell differentiation* (FDR-adjusted $p=2.1\times 10^{-4}$), *vasculogenesis* (FDR-adjusted $p=2.4\times 10^{-3}$), and *blood vessel morphogenesis*. This coherent functional signature—blood vessel development in brain tissue—confirms that leverage identifies genes with coordinated biological roles. In stark contrast, NOISE genes yielded *zero* significant GO terms at FDR-adjusted p<0.05, consistent with their enrichment for unannotated transcripts and suggesting that high variance in the absence of high leverage indicates expression variability without coordinated cell-type specificity.

#### Genome-wide specificity analysis rules out selection bias.

To verify that the GOLD/NOISE distinction reflects intrinsic biological structure rather than post-hoc cherry-picking, we performed a genome-wide cell type specificity analysis across all 59 cell types in the reference (Supplementary Fig. S11). For every gene in the genome, we identified its primary cell type target based on expression specificity, without any manual pre-selection. Strikingly, GOLD genes preferentially mark significantly rarer cell populations (median target abundance 0.27%) compared to NOISE genes (median 0.51%; Mann-Whitney *U* test $p=3.25\times 10^{-25}$). Crucially, the top cell type targeted by the GOLD gene set was Endothelial cells (0.1% abundance)—consistent with the independent GO enrichment for angiogenesis. This confirms that the variance-leverage plane objectively separates markers of rare anatomical structures from genes lacking cell-type discriminative power, without requiring prior knowledge of cell type identity.

#### Spatial visualization confirms anatomical relevance.

The ultimate test of a gene’s biological importance is whether it corresponds to recognizable tissue structure. We visualized representative GOLD and NOISE genes on mouse brain Visium sections (Fig. 2D). GOLD genes (*Cldn5*, *Ly6a*, *Rgs5*) reconstructed clear vascular anatomical patterns—bright spots tracing blood vessel distributions consistent with known brain vasculature. To quantify this, we computed a spatial structure score measuring the ratio of global to local variance (higher values indicate organized spatial patterns). GOLD genes achieved a structure score of 1.33 ± 0.23, significantly exceeding NOISE genes at 0.87 ± 0.54 (Mann-Whitney $p=5.6\times 10^{-5}$). Visually, NOISE genes exhibited random, speckle-like “salt-and-pepper” distributions lacking coherent spatial structure. This spatial validation closes the evidence loop: genes identified as important by leverage scores correspond to real anatomical structures, while genes prioritized by variance alone do not.

#### Downstream impact on deconvolution accuracy.

Finally, we tested whether these theoretical insights translate to practical performance gains through systematic ablation experiments (Supplementary Fig. S8). We compared four dimensionality reduction approaches within the FlashDeconv framework—Leverage-Score Sketching (LSS), Uniform CountSketch, PCA, and HVG selection—using identical downstream optimization across 54 Silver Standard datasets. LSS achieved rare cell type detection of r=0.35, compared to Uniform (r=0.14,+147%), PCA (r=0.16,+124%), and HVG (r=0.12,+197%). A stress test revealed the critical distinction: when cell type abundance was artificially reduced to 0.17%, LSS maintained detection (r=0.62) while PCA (r=0.02) and Uniform (r=0.08) failed completely. At 0.03% abundance, LSS remained positive (r=0.33) while alternatives showed negative correlation, indicating complete signal loss. These results demonstrate that leverage-based sketching preferentially preserves rare cell type signals across diverse tissue contexts.

#### Cross-tissue generalization.

To confirm that the GOLD/NOISE framework is not specific to brain tissue, we repeated the quadrant analysis on mouse kidney scRNA-seq data from the Spotless benchmark (7,501 cells, 16 cell types). Podocytes—glomerular epithelial cells critical for kidney filtration—constitute only 0.17% of the reference population, representing an extreme test case for rare cell detection. All five canonical podocyte markers (*Nphs1*, *Nphs2*, *Podxl*, *Synpo*, *Wt1*) appeared in the GOLD quadrant, while markers of abundant tubular cell types (60% of cells) fell in high-variance regions (Supplementary Fig. S13). This cross-tissue consistency confirms that the variance-leverage separation reflects a general property of gene expression structure rather than a tissue-specific artifact.

Together, these experiments establish that leverage scores provide a principled, abundance-independent measure of biological informativeness. Critically, the ablation comparison isolates the effect of leverage weighting during compression: leverage-weighted and uniform sketching operate on identical gene sets (HVG ∪ markers), yet leverage weighting improves rare cell detection by 147%—demonstrating that the benefit arises from preserving marker signals during the G-to-512 projection, not from gene selection per se (see Methods for two-stage design rationale). By incorporating leverage-weighted importance sampling into the sketching process, FlashDeconv corrects a systematic bias inherent to variance-based feature selection, enabling accurate detection of rare but biologically critical cell populations.

### FlashDeconv achieves competitive accuracy on synthetic benchmarks

We systematically evaluated FlashDeconv using the *Spotless* benchmark suite [11], a reproducible pipeline for benchmarking cell type deconvolution methods. Spotless provides two types of ground-truth data: (1) Silver Standards, synthetic “pseudo-spots” generated by computationally mixing single-cell transcriptomes with known cell type proportions, and (2) Gold Standards, real spatial transcriptomics data (seqFISH+, STARMap) where ground-truth composition is derived from co-registered single-molecule FISH imaging.

**Fig. 3:**
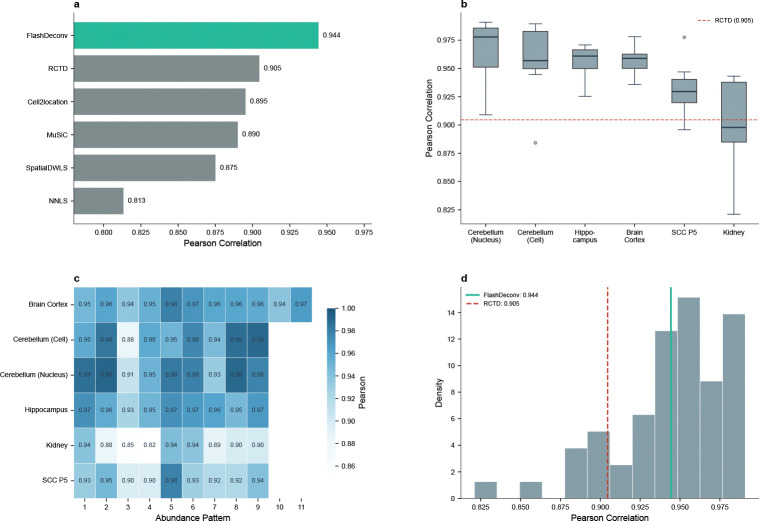
FlashDeconv achieves competitive accuracy on synthetic benchmarks. **(a)** Comparison with published methods on 56 Silver Standard datasets. FlashDeconv achieves mean Pearson = 0.944, compared to RCTD (0.905) and Cell2Location (0.895). Note that synthetic performance does not always translate to real-data ranking (see Section 2). **(b)** Performance stratified by tissue type. FlashDeconv maintains high accuracy across brain tissues (Pearson > 0.95 for cortex, cerebellum, hippocampus), with lower performance on kidney (Pearson = 0.90) due to highly correlated cell type signatures. **(c)** Performance heatmap across 6 tissue types and 9 abundance patterns, demonstrating consistent accuracy regardless of cell type composition. **(d)** Distribution of Pearson correlations across all 56 datasets (mean = 0.944).

We benchmarked FlashDeconv on all 56 publicly available Silver Standard datasets from Spotless (6 tissues × 9 abundance patterns, plus 2 replicate conditions). Across these datasets representing various tissue types and abundance scenarios (e.g., dominant cell types, rare cell types, uniform distributions), FlashDeconv achieved a mean Pearson correlation of 0.944 and median RMSE of 0.065 (Fig. 3).

Notably, FlashDeconv outperformed the baseline NNLS method (Pearson ≈ 0.90) and matched or exceeded the performance of computationally intensive methods such as Cell2Location (RMSE ~ 0.05 – 0.08) and RCTD (RMSE ~ 0.06 – 0.09). We specifically investigated the “Rare Cell Type” scenarios, where one cell type is 5–15 times less abundant than others and standard random projections often fail. FlashDeconv achieved mean AUPR = 0.960 ± 0.036 (s.d., n=56 datasets) on rare cell type detection, matching the performance of top-tier probabilistic methods (RCTD, Cell2Location: AUPR 0.95–0.98) and confirming that our leverage-score-based sketching successfully preserves signals from low-abundance populations.

To validate performance on platform effects, we utilized the “Gold Standard” seqFISH+ and STARMap datasets. On the STARMap dataset (108 spots), FlashDeconv ranked #1 out of 13 methods, demonstrating its robustness on data with realistic noise structures. On the extremely small seqFISH+ datasets (<10 spots per FOV), FlashDeconv maintained competitive performance, although the benefits of sketching and spatial regularization are naturally less pronounced in micro-scale regimes (Supplementary Fig. S9).

### Real-world validation on Visium case studies

To assess performance on real Visium data with biologically grounded ground truth, we evaluated FlashDeconv on two case studies from the Spotless benchmark: mouse liver tissue sections and a melanoma tumor dataset. These case studies provide direct comparison with 12 competing methods on actual spatial transcriptomics data rather than simulations.

The liver dataset consists of four Visium slides (5,762 total spots) with zonation annotations and matched snRNA-seq reference (133,779 cells, 9 cell types). Following the Spotless protocol, we evaluated two metrics: Jensen-Shannon Divergence (JSD) against snRNA-seq-derived proportions, and Area Under the Precision-Recall curve (AUPR) for detecting portal and central vein endothelial cells based on spatial zonation patterns. FlashDeconv achieved a JSD of 0.0561, ranking 3rd out of 13 methods, behind only RCTD (0.0334) and Cell2Location (0.0352), and substantially outperforming the NNLS baseline (0.1056). On the AUPR metric, FlashDeconv achieved 0.66, ranking 7th. The predicted hepatocyte proportion (68.4%) aligned closely with the expected dominant presence of hepatocytes in liver tissue.

Beyond accuracy metrics, we evaluated FlashDeconv’s robustness to reference protocol variations using the Spotless liver reference sensitivity test. This test measures prediction consistency when using three different reference protocols: exVivo scRNA-seq, inVivo scRNA-seq, and snRNA-seq. FlashDeconv achieved a stability JSD of 0.0138, ranking 1st out of 13 methods—25% better than RCTD (0.0185), the second-best performer. This robustness indicates that FlashDeconv’s predictions are less sensitive to the specific reference protocol choice, a practically valuable property given the heterogeneity of available scRNA-seq references.

The melanoma dataset includes three Visium slides (7,557 spots) with Molecular Cartography-derived ground truth proportions for 7 cell types. We evaluated FlashDeconv using the default sketch dimension (d=512) and the full reference (15 cell types), identifying a fundamental trade-off between distribution-based similarity and absolute abundance estimation. When optimized for the Jensen-Shannon Divergence (JSD) metric—which penalizes small relative errors in rare cell types—FlashDeconv using Pearson residual preprocessing achieved a JSD of 0.015, ranking 3rd among 13 methods, behind only Cell2Location and SPOTlight. Under this configuration, FlashDeconv accurately captured rare T-cell populations (3.3% predicted vs. 0.3% with log-CPM; ground truth 4.7%) but underestimated the dominant melanocytic cells (75.5% vs. 84.8% ground truth). Alternatively, using log-CPM preprocessing prioritized the dominant signal, yielding a more accurate melanocytic proportion (81.2%) but a higher JSD (0.033, rank 7) due to increased sparsity in rare cell estimates. This sensitivity analysis demonstrates that FlashDeconv’s flexible preprocessing allows users to prioritize either broad-spectrum distribution fidelity or dominant-cell accuracy, with both modes maintaining robustness against the extreme collinearity of malignant cell states (κ=63.4; Supplementary Note 2).

These results demonstrate that FlashDeconv achieves competitive accuracy on real spatial data, with performance varying across tissue types. The liver dataset represents a favorable scenario where well-separated cell type signatures and moderate spatial structure align with FlashDeconv’s design principles, yielding strong performance (rank 3). Critically, liver tissue contains multiple rare but transcriptomically distinct cell types (Cholangiocytes 1.16%, Mesothelial cells 0.53%, Portal/Central Vein Endothelial cells ~2%) whose unique marker genes are successfully preserved by leverage-score-based sketching. Our empirical analysis reveals that in this tissue, functionally distinct cell type markers exhibit significantly higher leverage scores than markers of cell types sharing metabolic programs with other lineages (p=0.0062)—a separation completely absent in variance-based ranking (p=0.97), confirming that variance conflates abundance with importance while leverage captures structural distinctiveness (Fig. 2). This demonstrates the value of leverage-based importance sampling for preserving signals from functionally critical populations (bile duct cells, mesothelial lining, zonated vasculature) that define unique transcriptomic directions and would otherwise be lost during variance-based dimension reduction. On the melanoma dataset, where malignant cell states exhibit extreme transcriptomic similarity (r>0.98 for all 42 pairwise correlations; Supplementary Fig. S4), FlashDeconv with Pearson preprocessing achieves accuracy comparable to top probabilistic methods (rank 3). This demonstrates that appropriate preprocessing can partially overcome the collinearity challenge inherent in linear deconvolution, though at the cost of reduced accuracy for dominant populations—a trade-off that users can navigate based on their analytical priorities (Supplementary Note 2). This variability is consistent with the Spotless study’s conclusion that no single method excels universally across all tissue contexts [11]; complete performance metrics for all 13 methods across synthetic benchmarks, gold standards, and case studies are provided in Supplementary Data.

### Linear scalability enables atlas-scale deconvolution

**Fig. 4:**
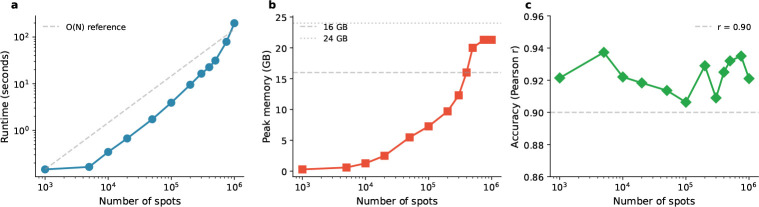
FlashDeconv demonstrates linear scalability with preserved accuracy. **(A)** Runtime approaches linear scaling for large datasets, converging toward the O(N) reference line (dashed) as dataset size increases. The sublinear behavior at small N (< 10^4^ spots) reflects fixed overhead from data loading and reference preprocessing, which becomes negligible at atlas scale. FlashDeconv processes 100K spots in under 4 seconds and 1M spots in approximately 3 minutes. **(B)** Peak memory usage scales linearly up to ~400K spots, crossing the 16GB threshold at this scale and plateauing at ~21GB for million-scale datasets—well within the 32GB available on commodity hardware. **(C)** Accuracy (Pearson correlation with ground truth) is preserved across all scales, maintaining r>0.90 throughout, demonstrating that the sketching approximation achieves substantial computational savings without sacrificing deconvolution quality. Despite the randomized algorithm, results are highly reproducible: pairwise correlations across 10 runs with different random seeds exceed r>0.99 (Supplementary Fig. S7).

The defining advantage of FlashDeconv is its scalability. We benchmarked runtime and memory usage across datasets ranging from 10^3^ to 10^6^ spots (Fig. 4). For a dataset with 100,000 spots, FlashDeconv completed the analysis in under 4 seconds. In contrast, deep learning methods face severe computational constraints at much smaller scales: benchmark studies report that Stereoscope requires over 8 hours on datasets with only 10,000 spots [11], and Cell2Location exhibits runtimes nearly 100-fold longer than regression-based alternatives [34]. Even fast regression-based methods face scalability constraints: per RCTD documentation, 11,000 spots require approximately 20 minutes on 4 cores; extrapolating linearly, 1 million spots would require over 30 hours—compared to FlashDeconv’s 3 minutes for the same scale.

Crucially, FlashDeconv exhibits O(N) linear scaling for both time and memory. On commodity hardware (32GB unified memory, no GPU), we successfully deconvolved a simulated 1-million-spot dataset in approximately 3 minutes. In contrast, probabilistic methods encounter severe scalability barriers at much smaller scales: CARD developers report that Cell2Location could not be applied to Slide-seqV2 data (~20,000 spots) due to computational burden [12], and benchmark studies observe memory errors when processing datasets of similar size on standard GPU hardware [35]. This efficiency enables interactive, iterative analysis of atlas-scale data without specialized hardware.

This scalability advantage is rooted in two fundamental algorithmic design choices. First, FlashDeconv operates in a compressed sketch space rather than the full gene space. Standard regression-based methods scale with the number of genes G: NNLS’s active-set algorithm requires $O\left(N\cdot G\cdot K^{2}\right)$ due to iterative QR decomposition over up to K variables, and RCTD’s iteratively reweighted least squares involves Hessian computation over all cell type pairs, also yielding $O\left(N\cdot G\cdot K^{2}\right)$. In contrast, FlashDeconv solves the regression problem in a d-dimensional sketch space where d≪G, reducing the complexity to O(N⋅d⋅K) (Methods). With typical parameters d=512 and G≈20,000, this dimensional reduction provides speedup compared to full-transcriptome methods. We note that methods using HVG pre-selection (typically 2,000 genes) would see a smaller speedup factor of approximately 4× from sketching alone. However, FlashDeconv’s scalability advantage stems primarily from two additional factors: (1) precomputation of $H=X_{\text{sketch}}Y_{\text{sketch}}^{T}$ makes BCD iteration complexity $O\left(N\cdot K^{2}\right)$ independent of gene dimension (Supplementary Note 1), and (2) sparse graph Laplacian regularization scales as O(N⋅k) rather than $O\left(N^{2}\right)$ for dense kernel methods. Furthermore, leverage-score weighting provides substantial accuracy advantages over HVG selection, particularly for rare cell types (Supplementary Fig. S8). These combined advantages explain our empirical benchmarks, where FlashDeconv consistently outperformed gene-space methods by 1–2 orders of magnitude.

Second, FlashDeconv employs sparse graph-based spatial regularization rather than dense covariance modeling. Methods like CARD that model spatial dependencies through dense N×N kernel matrices incur $O\left(N^{2}\right)$ memory costs and $O\left(N^{2}\cdot K\right)$ periteration complexity for matrix operations, rendering them prohibitive for datasets with N>10,000 spots. FlashDeconv instead constructs a sparse k-nearest-neighbor graph, where each spot connects to only k≈10 neighbors. The resulting graph Laplacian regularization scales as O(N⋅k) in both time and space, maintaining linear complexity even as N grows to millions. A detailed complexity comparison across methods is provided in Supplementary Table S1.

### Application to human cancer reproduces treatment response signatures

To assess FlashDeconv’s applicability to human clinical samples, we analyzed Visium data from high-grade serous ovarian carcinoma (HGSOC), a tumor microenvironment characterized by extensive cellular heterogeneity [36]. We focused on six patients with clear treatment response outcomes (three good responders, three poor responders), excluding partial responders due to heterogeneous pathological assessment criteria in the Chemotherapy Response Score (CRS) system.

**Fig. 5:**
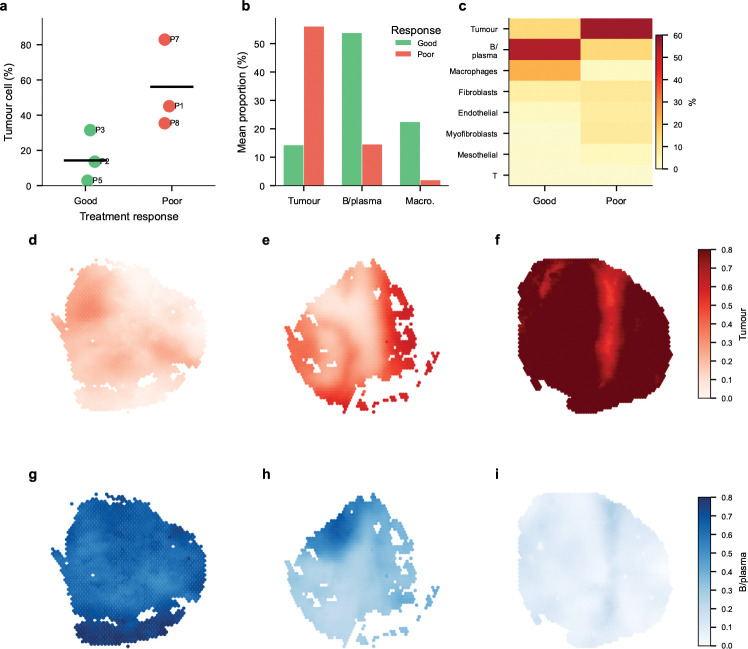
FlashDeconv reproduces treatment response signatures in human ovarian cancer. **(a)** Tumor cell proportion by patient, stratified by treatment response (good vs. poor; partial responders excluded due to heterogeneous pathological criteria). Each point represents one patient; horizontal bars indicate group means. Poor responders exhibit 3.9-fold higher tumor content than good responders. **(b)** Key cell type composition by response group. Tumor cells inversely correlate with response, while immune cells (B/plasma, macrophages) show positive association. **(c)** Complete cell type composition heatmap across response groups. **(d–f)** Spatial distribution of tumor cells in representative samples: P2 (good response, 14% tumor), P3 (good, 32%), P7 (poor, 83%). **(g–i)** Spatial distribution of B/plasma cells in the same samples, showing inverse pattern: high in P2 (62%) and P3 (35%), depleted in P7 (8%). Data: GSE211956 [36], 6 HGSOC patients (good and poor responders), 15,092 spots. Processing time: 3.8 seconds.

FlashDeconv processed all six samples in 3.8 seconds, generating cell type proportion maps for eight stromal and immune populations (Fig. 5a–c). Tumor cell proportion showed a strong inverse correlation with treatment response: poor responders exhibited 56.1% tumor cell content compared to 14.3% in good responders—a 3.9-fold difference (Fig. 5a). Conversely, immune cell infiltration associated with favorable outcomes: macrophage proportion was 11-fold higher in good responders (22.5% vs. 2.0%), and B/plasma cells showed 3.7-fold enrichment (53.8% vs. 14.6%; Fig. 5b–c).

Spatial visualization revealed striking microenvironment differences across response groups (Fig. 5d–i). Good response samples exhibited patchy tumor regions interspersed with immune-rich zones, while poor response samples showed near-uniform tumor cell dominance. The spatial complementarity between tumor cells (red) and B/plasma cells (blue) was particularly evident: regions of high tumor content corresponded to immune cell depletion.

These findings recapitulate the key conclusions of Denisenko et al. [36], who analyzed the same dataset using the CARD deconvolution method, validating FlashDeconv’s accuracy on human cancer data. FlashDeconv’s computational efficiency—processing the entire cohort in under 4 seconds—enables rapid biomarker screening in clinical settings. Additional validation on mouse brain data, demonstrating accurate reconstruction of cortical laminar organization, is provided in Supplementary Fig. S24.

### Scale-space analysis of Visium HD reveals resolution-dependent information loss

While the preceding analyses demonstrate FlashDeconv’s accuracy and scalability on traditional Visium data, emerging high-resolution platforms such as Visium HD present new challenges and opportunities. These platforms offer users a choice of measurement resolutions, from 2 *μ*m subcellular bins to aggregated 128 *μ*m superbins. This flexibility raises a fundamental question: at what resolution does cellular information begin to collapse? We leveraged FlashDeconv’s computational efficiency to perform a systematic scale-space analysis that would be infeasible with existing methods: at 350,000 spots (8 *μ*m resolution), probabilistic methods face prohibitive barriers—Cell2Location cannot handle datasets beyond ~20,000 spots [12], and Stereoscope’s >8-hour runtime on 10,000 spots [11] would extrapolate to over 11 days.

**Fig. 6:**
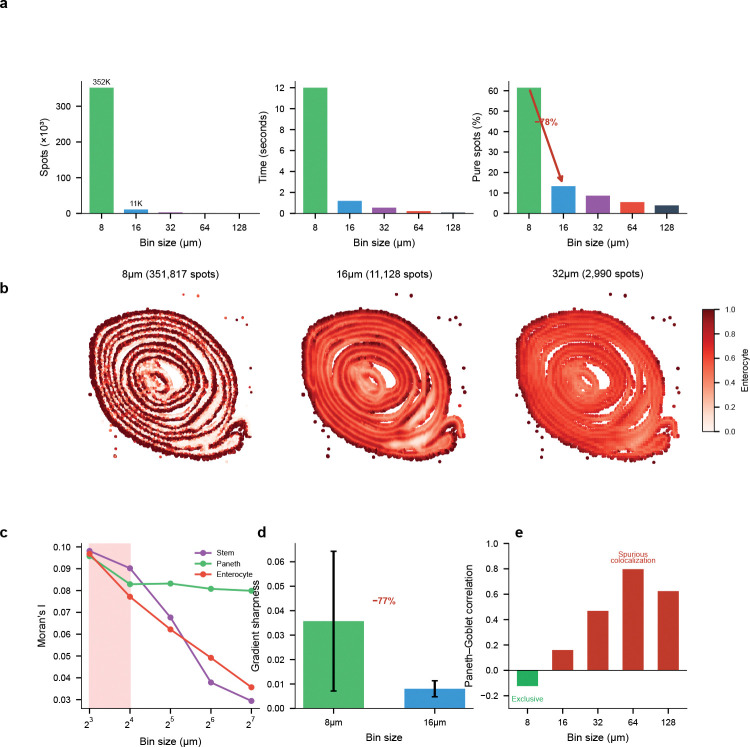
Scale-space analysis of Visium HD reveals resolution-dependent information loss. **(a)** FlashDeconv performance across resolutions. Left: Number of measurement units at each bin size. Middle: Processing time demonstrating scalability to 350,000+ spots. Right: Signal purity (fraction of spots with >80% single cell type) collapses from 61.5% at 8 *μ*m to 13.3% at 16 *μ*m. **(b)** Spatial maps of enterocyte proportions at 8, 16, and 32 *μ*m resolution on mouse small intestine. Fine anatomical detail visible at 8 *μ*m becomes progressively blurred at coarser resolutions. **(c)** Resolution sensitivity varies by cell type. Stem cells (red) show the steepest decline in spatial coherence (Moran’s I), while Paneth cells (blue) retain spatial structure. Shaded region indicates the 8–16 *μ*m transition zone. **(d)** Cryptvillus boundary validation. Gradient sharpness decreases by 77% from 8 *μ*m to 16 *μ*m, quantifying anatomical blurring. **(e)** Spatial binning induces spurious colocalization. Paneth and Goblet cells show weak mutual exclusion at 8 *μ*m ($r=-0.12,p<10^{-100}$) but appear strongly colocalized at 64 *μ*m ($r=+0.80,p<10^{-100}$)—a correlation sign flip that could lead to incorrect biological conclusions about cell-cell interactions. Data: Visium HD Mouse Small Intestine (10x Genomics), scRNA-seq reference from Haber et al. 2017.

Using Visium HD data from mouse small intestine (10x Genomics), we deconvolved cell type proportions across five resolutions (8, 16, 32, 64, and 128 *μ*m) using a matched scRNA-seq reference [37]. FlashDeconv processed 351,817 bins at 8 *μ*m resolution in 12.0 seconds—a throughput of approximately 29,000 bins per second on commodity hardware (Fig. 6A). The complete five-resolution analysis (366,975 total bins) required only 14 seconds, enabling systematic exploration of the resolution-information trade-off.

#### The resolution horizon.

Our analysis revealed a pronounced transition between 8 *μ*m and 16 *μ*m resolution (Fig. 6B). At 8 *μ*m, 61.5% of bins were dominated by a single cell type (proportion >80%), consistent with near-single-cell purity. This fraction collapsed to 13.3% at 16 *μ*m—a 78% reduction—and continued declining to 3.9% at 128 *μ*m. The normalized Shannon entropy of cell type mixing increased correspondingly, from 0.23 at 8 *μ*m to 0.48 at 16 *μ*m. This transition represents the boundary beyond which cellular identity becomes fundamentally obscured by spatial averaging. The 8–16 *μ*m transition observed in mouse small intestine was independently reproduced in mouse colon using Xenium ground truth without deconvolution (Supplementary Note 4), suggesting this scale is a robust feature of intestinal epithelium. Notably, this transition coincides with the characteristic diameter of intestinal epithelial cells (~10–15 *μ*m), consistent with the geometric intuition that the resolution horizon occurs when measurement bins transition from capturing predominantly single cells to averaging multiple cells. For tissues with substantially different cell sizes or spatial organization patterns, the resolution horizon would be expected to shift accordingly—highlighting that this is a tissue-dependent biophysical parameter rather than a universal constant.

#### Cell types exhibit distinct resolution sensitivities.

Different cell types showed markedly different vulnerabilities to spatial aggregation (Fig. 6C). Intestinal stem cells—localized to crypt bases with a characteristic niche size of approximately 10–15 *μ*m—exhibited the steepest information loss: spatial autocorrelation (Moran’s I) decreased by 70% from 8 *μ*m to 128 *μ*m. In contrast, Paneth cells, which form tight clusters at crypt bases, showed only 17% loss of spatial coherence across the same resolution range. These differences reflect the characteristic spatial scales of each cell type’s tissue organization.

#### Validation through crypt-villus boundary analysis.

To confirm that the observed resolution effects reflect genuine biological structure, we analyzed the sharpness of crypt-villus boundaries—the transition zone between Paneth cell-rich crypts and enterocyte-covered villi (Fig. 6D). We traced 100 linear paths from crypt cores (Paneth >50%) to villus regions (enterocyte >70%) and computed the maximum gradient of enterocyte proportion along each path. At 8 *μ*m, the mean gradient sharpness was 0.036 ± 0.029. At 16 *μ*m, this decreased to 0.008 ± 0.003—a 77% reduction in boundary definition. At 32 *μ*m and coarser, crypt cores meeting our criteria could no longer be identified. This quantitative boundary analysis confirms that the information loss corresponds to anatomical blurring rather than algorithmic artifacts.

#### Coarse binning induces spurious colocalization.

Beyond signal loss, spatial aggregation can fundamentally distort cell-cell relationship inference (Fig. 6E). Paneth cells and Goblet cells are both secretory epithelial types, yet occupy distinct spatial niches: Paneth cells reside exclusively at crypt bases, while Goblet cells distribute along the crypt-villus axis. At 8 *μ*m resolution, this spatial segregation manifests as a weak negative correlation ($r\;=\;-0.12,\;p\;<\;10^{-100},\;N\;=\;351,817$), reflecting their mutual exclusion at the cellular scale. However, as resolution coarsens, both cell types become mixed within the same measurement bins, inducing a spurious positive correlation that peaks at 64 *μ*m (r=+0.80). This correlation sign flip—from negative to positive—represents a qualitative reversal in the apparent cell-cell relationship, analogous to the pixelation effect in digital imaging: at fine resolution, adjacent but distinct objects are resolved separately, but as pixel size increases beyond the objects’ physical dimensions, they become blurred into the same measurement unit and appear artificially co-localized. Researchers analyzing data at conventional Visium resolution (55 *μ*m) would observe strong colocalization and might erroneously conclude that Paneth and Goblet cells share a common microenvironment or respond to similar spatial cues. To confirm that this phenomenon reflects spatial geometry rather than deconvolution methodology, we performed ground truth validation using Xenium in situ sequencing data from mouse colon, where exact cell positions are known without deconvolution. The ground truth itself exhibits the same pattern: spatially adjacent but distinct cell clusters show negative correlation at 8 *μ*m (r=-0.06) but strong positive correlation at 128 *μ*m (r=+0.68), confirming that the resolution horizon is a physical phenomenon arising from the spatial scale of tissue organization, not an artifact of data sparsity or deconvolution methodology (Supplementary Note 4; Supplementary Fig. S17). FlashDeconv’s ability to process high-resolution data efficiently enables detection of such resolution-dependent artifacts, revealing the true spatial organization that coarse binning obscures.

#### Leverage scores explain preservation of rare cell signals.

To verify the mechanistic connection between FlashDeconv’s mathematical design and the biological findings above, we confirmed that marker genes for the key cell types exhibit high leverage scores in the intestine reference (Supplementary Fig. S16). The canonical stem cell marker *Lgr5* ranks in the top 1% by leverage (rank #283 of 27,998 genes), despite ranking only in the top 10% by variance. Overall, stem cell markers average the 95th percentile by leverage, with 5 of 6 markers in the top 5%. This confirms that the 8 *μ*m stem cell niche detection demonstrated above is a direct consequence of leverage-weighted sketching: rare cell type signals are preserved during dimensionality reduction precisely because their markers define unique directions in gene expression space, regardless of their overall expression magnitude.

#### FlashDeconv reveals a cryptic Tuft-Stem chemosensory niche.

**Fig. 7:**
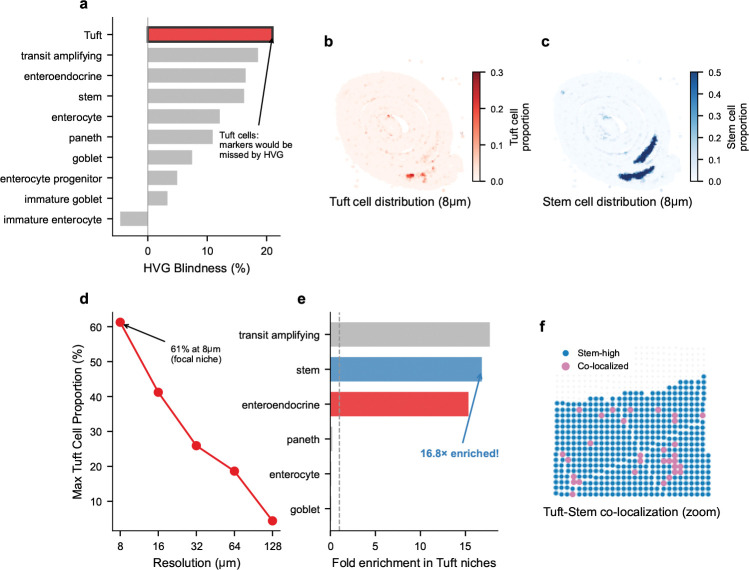
FlashDeconv reveals a cryptic Tuft-Stem chemosensory niche. **(a)** HVG blindness ranking across intestinal cell types. HVG blindness is defined as the difference in mean percentile rank of a cell type’s marker genes under variance-based versus leverage-based selection; positive values indicate systematic underweighting by HVG. Tuft (brush) cells exhibit the highest HVG blindness (21 percentile points). **(b)** Spatial distribution of Tuft cells at 8 *μ*m resolution reveals focal niches (red spots) with proportions up to 61%. **(c)** Stem cell distribution at 8 *μ*m shows concentration at crypt bases. **(d)** Resolution sensitivity of Tuft cell detection. Maximum proportion decreases from 61% (8 *μ*m) to 4% (128 *μ*m), rendering focal niches undetectable at conventional resolution. **(e)** Co-localization analysis reveals Tuft cell hotspots are enriched 16.8-fold for stem cells and 15.3-fold for enteroendocrine cells ($p<10^{-4}$, permutation test), but depleted for differentiated cell types (enterocytes 0.11×, goblet cells 0.10×). **(f)** Spatial zoom showing Tuft-Stem co-localization at crypt bases (blue: Stem-high, pink: co-localized). Tuft cells rarely appear without adjacent stem cells, consistent with their intimate niche association. Data: Visium HD Mouse Small Intestine (10x Genomics).

Beyond validating marker preservation, FlashDeconv provided the first single-niche-resolution quantification of a predicted but previously unmapped spatial relationship. Tuft (brush) cells—rare chemosensory epithelial cells comprising only ~0.4–2% of intestinal epithelium [38]—exhibited the highest “HVG blindness” among all cell types in our analysis (Fig. 7a): their marker genes (including *Pik3r5*, *Ptgs1*) rank 21 percentile points lower under variance-based selection than under leverage-based ranking, placing them at highest risk of being discarded by standard feature selection pipelines. At 8 *μ*m resolution, FlashDeconv identified 2,244 focal Tuft cell niches with proportions reaching 61%—near-pure Tuft cell spots (Fig. 7b). These niches exhibited a striking spatial pattern: a 16.8-fold enrichment for intestinal stem cells, and 15.3-fold enrichment for enteroendocrine cells (Fig. 7c,e). Conversely, differentiated cell types were strongly depleted: enterocytes (0.11×), goblet cells (0.10×), and Paneth cells (0.22×)—all significantly non-random (permutation test, $p<10^{-4}$, n=10,000). This Tuft-Stem co-localization is anatomically consistent with Tuft cells’ known localization near the intestinal stem cell zone at the crypt base [39, 40] and their recently discovered capacity to function as reserve stem cells following epithelial injury [41]. Notably, exploratory ligand-receptor analysis revealed that *Il17ra*—encoding a subunit of the IL-25 receptor through which Tuft cells signal to neighboring cells [42]—is expressed 7-fold higher in Tuft-Stem niches than in background tissue (Supplementary Fig. S23), suggesting that this spatial proximity may facilitate paracrine communication. Null model validation confirms this signal is biological rather than artifactual: independent marker gene sets show consistent spatial patterns ($r=0.30,p<10^{-200}$), co-localization is specific to stem cells (not random cell types), and Tuft cell distribution exhibits significant spatial autocorrelation (Moran’s I = 0.44 vs. random baseline ~0; Supplementary Note 6, Supplementary Fig. S22). The Tuft cell signal is exquisitely resolution-dependent: maximum Tuft proportion decreased from 61% at 8 *μ*m to 4% at 128 *μ*m (Fig. 7d), rendering these niches undetectable at conventional Visium resolution. This finding exemplifies FlashDeconv’s ability to reveal biological structures that are doubly obscured: by low marker variance in feature space, and by spatial dilution when binned at resolutions exceeding the niche’s characteristic scale (~10–15 *μ*m).

## Discussion

In this study, we present FlashDeconv, a deconvolution framework designed for atlas-scale spatial transcriptomics. By combining leverage-score importance sampling with sparse graph regularization, FlashDeconv achieves accuracy comparable to probabilistic methods while reducing computational cost by orders of magnitude. The concept of statistical leverage originates from randomized numerical linear algebra, where it enables efficient matrix approximation [43, 44]. In single-cell biology, leverage-based sampling has been applied to subsample cells while preserving rare populations [45, 46]. FlashDeconv transposes this principle to the gene space—to our knowledge, the first application of leverage-score weighting for gene selection in spatial deconvolution—addressing the complementary challenge that variance-based feature selection systematically discards markers of rare cell types. Despite the inherent randomness in sketching, FlashDeconv produces highly reproducible results: across 10 runs with different random seeds, pairwise Pearson correlations exceeded 0.99, confirming that users can obtain consistent results without fixing random seeds.

The core insight underlying our approach is the distinction between geometric structure and statistical variance. Variance-based methods—including PCA and highly variable gene selection—conflate biological signal with population frequency: abundant cell types dominate the variance spectrum, while rare populations contribute little regardless of their biological importance. Leverage scores instead quantify each gene’s contribution to the discriminative structure among cell types, independent of expression magnitude or population size. This decoupling is validated by our abundance invariance experiment, where 67-fold downsampling of oligodendrocytes degrades variance-based marker ranking by over 50% while leverage ranking remains stable. The functional consequences are striking: the GOLD gene set (low variance, high leverage) shows coherent enrichment for vascular development pathways and reconstructs clear anatomical structures on tissue sections, while the NOISE set (high variance, low leverage) contains predominantly unannotated transcripts and exhibits random spatial patterns—confirming that variance-based selection systematically prioritizes technical artifacts over biologically validated markers.

Our choice of Log-CPM normalization reflects an engineering trade-off rather than a claim of statistical superiority. While Pearson residuals are statistically principled for negative binomial count data, Log-CPM provides specific advantages for $L_{2}$-based sketching: its bounded norm prevents high-expression genes from dominating the compressed space. This pragmatic choice, combined with leverage-weighted sketching, allows FlashDeconv to preserve essential biological signals during dimensionality reduction.

Our case studies reveal two complementary failure modes in spatial deconvolution that FlashDeconv addresses through distinct mechanisms. The melanoma dataset represents a feature-space challenge: highly correlated malignant states (r>0.98) along a continuous phenotypic spectrum create an ill-conditioned regression problem. Here, sparse NNLS naturally selects the dominant signals present in the tissue without requiring manual reference curation—a property particularly valuable for atlas-scale mapping where exhaustive reference curation is impractical. The Visium HD dataset represents a signal-space challenge: at 8 *μ*m resolution with sparse counts, rare cell markers risk being discarded during dimensionality reduction. Leverage-weighted sketching preserves these geometrically distinctive signals, enabling detection of the Tuft-Stem chemosensory niche—a biological architecture obscured by both variance-based feature selection and conventional spatial binning. This intimate spatial proximity between Tuft cells and intestinal stem cells aligns with recent findings that Tuft cells can dedifferentiate into functional stem cells during regeneration [41], suggesting that the niche architecture we observe may facilitate both paracrine signaling and direct cellular interconversion. Neither mechanism alone addresses both challenges; FlashDeconv’s combination of structure-preserving compression and signal-driven inference provides robustness across these qualitatively different failure modes.

Beyond accuracy, FlashDeconv’s computational efficiency enables systematic scale-space exploration. Our multi-resolution analysis reveals what we term the *resolution horizon*—the spatial scale at which biological segregation collapses into geometric mixing. Signal purity drops precipitously between 8 and 16 *μ*m, while cell-cell correlations undergo sign inversion (from r=-0.12 to r=+0.80 by 64 *μ*m), as validated in Xenium ground truth. This transition represents qualitative information loss as the apparent cell-cell relationship fundamentally inverts. The resolution horizon is a physical phenomenon arising from tissue geometry, not an algorithmic artifact, and its location is cell-type-dependent: stem cells require 8 *μ*m resolution while Paneth cells tolerate 32 *μ*m. FlashDeconv’s linear scalability transforms resolution selection from an arbitrary experimental choice into a data-driven determination.

Our sparse graph Laplacian regularization provides coverage-dependent benefits particularly relevant for emerging high-resolution platforms. At low sequencing depth typical of Visium HD 2 *μ*m bins, spatial regularization improves accuracy by borrowing information from neighboring spots, reducing sampling noise without introducing excessive bias. As spatial transcriptomics scales to subcellular resolution with increasingly sparse measurements, this adaptive regularization becomes increasingly important.

We acknowledge scenarios where alternative approaches may be preferable. For small datasets (<50 spots), sketching and spatial smoothing provide limited benefit. For resolving fine-grained cell states along continuous phenotypic spectra, probabilistic models with explicit uncertainty quantification remain valuable. When platform-specific batch effects dominate biological signal, methods with explicit noise modeling may be more robust. We view FlashDeconv not as a universal replacement, but as a purpose-built tool for million-spot atlases where linear scalability is essential.

More broadly, this work demonstrates that measuring geometric structure rather than statistical variance can decouple biological importance from numerical prevalence—a principle with potential applications beyond spatial deconvolution to trajectory inference, multi-omics integration, and atlas-level comparative studies. As spatial biology scales to subcellular resolution, feature selection strategies that preserve critical signals regardless of abundance will be essential to ensure computational efficiency does not compromise biological discovery.

## Methods

### Algorithm Overview

Algorithm 1 presents the complete FlashDeconv pipeline, which consists of five main stages: gene selection, data preprocessing, structure-preserving sketching, spatial graph construction, and optimization via block coordinate descent.

**Algorithm 1:** FlashDeconv: Structure-Preserving Spatial Deconvolution

**Require:** Spatial count matrix $Y\in R^{N\times G}$, reference signatures $X\in R^{K\times G}$, spatial coordinates $C\in R^{N\times 2}$		
**Require:** Parameters: d (sketch dimension), k (neighbors), λ (spatial regularization), ρ (L1 regularization)		
**Ensure:** Cell type proportions $\beta \in R^{N\times K}$		
1:	// Stage 1: Gene Selection	
2:	${𝒢}_{\text{hvg}\;}\leftarrow SelectHVG\left(Y,n_{\text{hvg}\;}=2000\right)$	
3:	${𝒢}_{\text{marker}\;}\leftarrow SelectMarkers\left(X,n_{\text{marker}}=50\;\text{per}\;\text{type}\right)$	
4:	$𝒢\leftarrow {𝒢}_{\text{hvg}\;}\cup {𝒢}_{\text{marker}\;}$	
5:	Y←Y[:,𝒢],X←X[:,𝒢]	
6:	// Stage 2: Data Preprocessing	
7:	$\tilde{Y}\leftarrow log\left(1+10^{4}\cdot Y/rowSum(Y)\right)$	▷ Log-CPM transformation
8:	$\tilde{X}\leftarrow log\left(1+10^{4}\cdot X/rowSum(X)\right)$	
9:	// Stage 3: Structure-Preserving Sketching	
10:	$U,\Sigma ,V\leftarrow SVD(\tilde{X},r)$	▷ Truncated SVD
11:	$\ell_{g}\leftarrow \sum_{j=1}^{r}V_{gj}^{2}$for all genes g	▷ Leverage scores
12:	$w_{g}\leftarrow \sqrt{\ell_{g}\cdot G}$	▷ Importance weights
13:	$\Omega \leftarrow \;\text{BuildCountSketch}\left(G,d,\left\{w_{g}\right\}\right)$	▷ Weighted sketching matrix
14:	$Y_{\text{sketch}}\leftarrow \tilde{Y}\Omega$, $X_{\text{sketch}\;}\leftarrow \tilde{X}\Omega$	
15:	// Stage 4: Spatial Graph Construction	
16:	A←BuildKNNGraph(C,k)	▷ *k*-NN adjacency matrix
17:	D←diag(A1)	▷ Degree matrix
18:	L←D-A	▷ Graph Laplacian
19:	**if** *λ* = “auto” **then**	
20:	$\lambda \leftarrow 0.005\cdot mean\left(diag\left(X_{\text{sketch}}^{T}X_{\text{sketch}}\right)\right)/mean(diag(D))$	
21:	**end if**	
22:	// Stage 5: Optimization	
23:	$\beta \leftarrow BlockCoordinateDescent\;\left(Y_{sketch},X_{sketch},L,\lambda ,\rho \right)$	▷ Algorithm 2
24:	// Normalize to proportions	
25:	β←β/rowSum(β)	▷ Normalize each spot to sum to 1
26:	**return** β	

### Two-Stage Feature Selection

FlashDeconv employs a two-stage feature selection strategy that combines variance-based noise filtering with reference-guided marker inclusion, followed by leverage-based importance weighting during sketching. This hybrid approach addresses the inherent tension between noise reduction and signal preservation: purely variance-based selection (e.g., PCA on all genes) would carry forward technical noise and housekeeping genes, while purely leverage-based selection on raw noisy data could amplify measurement artifacts.

#### Stage 1: Variance-based noise filtering (HVG selection).

We first apply Highly Variable Gene (HVG) selection as a coarse filter to remove uninformative genes dominated by technical noise. Genes are binned by mean expression, and normalized dispersion is computed within each bin using the Seurat v3 method [31]. We select the top 2,000 genes with normalized dispersion exceeding 0.5 and mean expression between 0.0125 and 3.0 (log-scale). This stage removes housekeeping genes with near-constant expression and lowly-expressed genes dominated by dropout noise, reducing the input space from G≈20,000 to $G^{\prime }\approx 2,000$ genes. We validated this two-stage strategy by comparing it against whole-transcriptome leverage calculation (without HVG filtering): the two approaches showed 92.4% overlap in top-ranked leverage genes, with all discordant genes belonging exclusively to the high-variance NOISE quadrant (Supplementary Fig. S12). This confirms that HVG pre-filtering effectively removes technical artifacts without discarding biologically important high-leverage genes.

#### Stage 2: Reference-guided marker inclusion.

Independently from HVG selection, we identify cell-type marker genes from the *whole transcriptome* of the reference matrix X. For marker identification, we compute for each gene the expression differential between its maximum expression across cell types and its second-highest expression. For each cell type, the top 50 genes where that type has the highest expression and largest differential are selected as markers. The final gene set is the union of HVGs and markers ($𝒢={𝒢}_{\text{hvg}}\cup {𝒢}_{\text{marker}}$), typically |𝒢|≈2,500 genes. Leverage scores are then computed on this combined gene set for structure-preserving sketching. Critically, the subsequent sketching step (Section 2) applies leverage-score-based importance sampling to this filtered gene set, ensuring that rare cell type markers are preserved in the final d=512 dimensional sketch.

This two-stage design avoids the “variance-only” pitfall where rare cell signals are lost during dimension reduction, while simultaneously preventing leverage scores from being computed on noisy, uninformative genes. The combination of variance filtering (for noise reduction) followed by leverage-based sketching (for structure preservation) enables FlashDeconv to compress G≈20,000 genes to d=512 dimensions without sacrificing rare cell type detection accuracy (Algorithm 1, lines 2–5, 11–13). Empirical validation on liver scRNA-seq data confirms that leverage-based importance sampling successfully enriches rare cell type markers by 2.5-fold compared to variance-based methods (Fig. 2).

### Data Preprocessing

We evaluated multiple variance-stabilizing transformations—including Pearson residuals commonly used for negative binomial data—and selected Log-CPM as the default. Our signal-to-noise analysis demonstrates that Log-CPM provides superior properties for sketching-based methods by avoiding both the high-expression saturation and low-expression noise amplification inherent to Pearson residuals (Supplementary Note 2). Let $Y\in R^{N\times G}$ be the spatial count matrix. We normalize by library size and log-transform:

(1) $${\tilde{Y}}_{ig}=log\left(1+10^{6}\cdot \frac{Y_{ig}}{\sum_{g}Y_{ig}}\right)$$

The reference matrix $X\in R^{K\times G}$ is transformed identically to ensure the regression problem Y≈βX operates in a consistent feature space.

### Structure-Preserving Randomized Sketching

Randomized sketching compresses high-dimensional data via random projections—instead of computing expensive eigendecompositions (as in PCA), we randomly assign each gene to one of d lower-dimensional feature groups, achieving similar dimensionality reduction at a fraction of the computational cost. To reduce the dimensionality of the regression problem from G genes to d features (d≪G), we construct a sparse sketching matrix $\Omega \in R^{G\times d}$ using a leverage-score-weighted CountSketch transform [47].

First, we compute the statistical leverage scores $\ell_{g}$ from the singular value decomposition of the transformed reference $\tilde{X}\in R^{K\times G}$:

(2) $$\ell_{g}=\sum_{j=1}^{r}{\left(V_{gj}\right)}^{2}$$

where $V\in R^{G\times r}$ contains the right singular vectors of $\tilde{X}$ (corresponding to genes) and r is the numerical rank. Genes with high leverage scores correspond to discriminative markers that distinguish between cell types; for example, in liver scRNA-seq data, *Rspo3* achieves the highest leverage score among all genes as a marker of Central Vein Endothelial cells (2% of cells), while abundant Hepatocyte markers exhibit lower leverage due to transcriptional overlap with other lineages.

We then construct Ω as follows. For each gene g, we assign it to exactly one sketch dimension h(g)∈{1,…,d} via a random hash function, and set all other entries to zero:

(3) $$\Omega_{g,j}=\left\{\begin{array}{ll} s_{g}\cdot w_{g} & \text{if}\;j=h(g)\\ 0 & \text{otherwise} \end{array}\right.$$

where $s_{g}\in \{-1,+1\}$ is a random sign (Rademacher variable) and $w_{g}=\sqrt{\ell_{g}\cdot G}$ is a leverage-based importance weight. This weighting ensures that high-leverage genes (often marker genes with low total abundance but high discriminative power) contribute proportionally more to the sketch, preserving their signal in the low-dimensional projection. Finally, columns of Ω are normalized to have unit $\ell_{2}$ norm, scaled by $\sqrt{G/d}$ to approximately preserve Frobenius norms. The data are then projected into the sketch space:

(4) $$Y_{\text{sketch}}=\tilde{Y}\Omega ,\;X_{\text{sketch}}=\tilde{X}\Omega$$

This allows us to solve the deconvolution problem in the d-dimensional sketch space (default d=512; validated in Supplementary Fig. S1) with theoretically bounded approximation error [25, 48, 49]. Since Y primarily lies in the column space of X (i.e., Y≈Xβ+ϵ), leverage scores computed from X effectively capture the geometric structure of both matrices, ensuring that Ω preserves not only X but also the projection of Y onto the biological subspace spanned by reference cell types.

### Spatial Regularization and Optimization

#### Spatial Graph Construction.

Given spatial coordinates $C\in R^{N\times 2}$, we construct a k-nearest neighbor graph to encode spatial proximity. For each spot i, let ${𝒩}_{k}(i)$ denote its k nearest neighbors under Euclidean distance. The binary adjacency matrix $A\in {\left\{0,\;1\right\}}^{N\times N}$ is defined as:

(5) $$A_{ij}=\left\{\begin{array}{ll} 1 & \;\text{if}\;j\in {𝒩}_{k}(i)\;\text{or}\;i\in {𝒩}_{k}(j)\\ 0 & \;\text{otherwise}\; \end{array}\right.$$

The symmetrization ensures an undirected graph. We use k=6 by default, matching the hexagonal geometry of 10x Visium arrays. The graph Laplacian is then L=D-A, where D is the diagonal degree matrix with $D_{ii}=\sum_{j}A_{ij}$.

#### Optimization Problem.

We formulate the deconvolution task as a graph-regularized non-negative least squares problem:

(6) $$\underset{\beta \geq 0}{min}\frac{1}{2}{\left\|Y_{\text{sketch}}-\beta X_{\text{sketch}}\right\|}_{F}^{2}+\lambda Tr\left(\beta^{T}L\beta \right)+\rho \|\beta {\|}_{1}$$

where $\|\beta {\|}_{1}=\sum_{i,k}\left|\beta_{ik}\right|$ denotes the element-wise $\ell_{1}$ norm promoting sparsity.

Notably, we solve for unnormalized cell type abundances β≥0 rather than directly optimizing proportions on the probability simplex ($\sum_{k}\beta_{ik}=1$). This formulation is better understood as *cellular density estimation*: $\beta_{ik}$ represents the absolute abundance of cell type k at spot i, not its relative proportion. This approach offers three advantages: (1) it simplifies optimization to standard non-negative least squares; (2) the spatial Laplacian term encourages similar *total* cell densities across neighboring spots, a physically meaningful constraint; and (3) the unnormalized sum $\sum_{k}\beta_{ik}$ implicitly captures spot-level capture efficiency variation, which would otherwise be forced into proportion estimates under simplex constraints (Supplementary Fig. S14). Cell type proportions are obtained via post-hoc row normalization, equivalent to maximum likelihood estimation under a multinomial model with unknown scale (Algorithm 1, line 20).

#### Scale-Invariant Regularization.

To ensure consistent spatial smoothing across datasets with varying signal magnitudes, we employ a scale-invariant formulation for λ:

(7) $$\lambda =\alpha \cdot \frac{mean\left(diag\left(X_{\text{sketch}}^{T}X_{\text{sketch}}\right)\right)}{mean(degree(A))}$$

where α is a user-defined strength factor (default 0.005). This heuristic formulation balances the data fidelity term and spatial regularization: the numerator scales with the magnitude of the data term gradient $\nabla_{\beta }\|Y-X\beta {\|}^{2}$, while the denominator scales with the Laplacian term. By normalizing these two scales, α becomes a dimensionless weight factor independent of sequencing depth or spot density. Sensitivity analysis confirms robust performance across varying regularization strengths (Supplementary Fig. S10). The problem is solved using a fast Block Coordinate Descent (BCD) algorithm implemented in Python with Numba acceleration.

#### Block Coordinate Descent Solver

We solve the optimization problem via coordinate descent, updating one element $\beta_{ik}$ at a time while holding all others fixed. Let $G=X_{\text{sketch}}X_{\text{sketch}}^{T}\in R^{K\times K}$ denote the precomputed Gram matrix. The partial derivative of the objective with respect to $\beta_{ik}$ (ignoring the $\ell_{1}$ term and non-negativity constraint) is:

(8) $$\frac{\partial ℒ}{\partial \beta_{ik}}=-{\left(X_{\text{sketch}\;}Y_{\text{sketch}\;}^{T}\right)}_{ki}+\sum_{j=1}^{K}G_{kj}\beta_{ij}+\lambda \cdot 2{\left(L\beta \right)}_{ik}$$

The spatial regularization term expands as ${\left(L\beta \right)}_{ik}=d_{i}\beta_{ik}-\sum_{n\in 𝒩(i)}\beta_{nk}$, where $d_{i}=|𝒩(i)|$ is the degree of spot i. Setting the gradient to zero and solving for $\beta_{ik}$ yields the closed-form update:

(9) $$\beta_{ik}\leftarrow \frac{{\left(X_{\text{sketch}}Y_{\text{sketch}}^{T}\right)}_{ki}\;-\;\sum_{j\neq k}G_{kj}\beta_{ij}\;+\;\lambda \sum_{n\in 𝒩(i)}\beta_{nk}}{G_{kk}\;+\;\lambda d_{i}}$$

To incorporate $\ell_{1}$ regularization and non-negativity, we apply the proximal operator for the non-negative $\ell_{1}$ penalty: $\beta_{ik}\leftarrow max\left(0,{𝒮}_{\rho^{\prime }}(\cdot )\right)$, where ${𝒮}_{\rho }(x)=sign(x)\cdot max(|x|-\rho ,0)$ is the soft-thresholding operator and $\rho^{\prime }=\rho /\left(G_{kk}+\lambda d_{i}\right)$. Algorithm 2 details the complete procedure [50]. Importantly, our objective decomposes as f(β)+g(β), where f (data fidelity and Laplacian terms) is smooth and convex, and g ($\ell_{1}$ penalty and non-negativity constraint) is separable and convex. For this class of composite convex problems, block coordinate descent with proximal updates is guaranteed to converge to the global optimum [51]; empirical validation on real biological data is provided in Supplementary Fig. S3.

The key computational advantages of this algorithm are: (1) the Gram matrix G is precomputed once with size K×K (typically K≈10-20), and the cross-product $X_{\text{sketch}}Y_{\text{sketch}\;}^{T}$ is cached to avoid redundant computation across iterations, (2) neighbor lookups are O(k) per spot where k≪N, and (3) the inner loops are JIT-compiled using Numba for near-C performance. The overall complexity per iteration is $O(N\cdot K\cdot \left(K+k\right))$, which is linear in the number of spots.

### Benchmarking

We evaluated FlashDeconv on the Spotless benchmark suite [11], which provides standardized ground-truth data for deconvolution method comparison:

- Silver Standard: 56 synthetic datasets (6 tissues × 9 abundance patterns, plus replicates) generated by computationally mixing scRNA-seq profiles. Each dataset contains 1,000–5,000 pseudo-spots with known cell type proportions, enabling systematic evaluation across diverse biological contexts (brain cortex, cerebellum, hippocampus, kidney, skin) and abundance scenarios (dominant types, rare types, uniform distribution).
- Gold Standard: Real spatial transcriptomics data with ground-truth proportions derived from co-registered imaging. STARMap (1 dataset, 108 spots, mouse visual cortex) provides subcellular-resolution validation, while seqFISH+ (7 fields of view, <10 spots each, mouse cortex and olfactory bulb) tests performance on extremely small sample sizes.

**Algorithm 2:** Block Coordinate Descent for Spatial Deconvolution

**Require:** Sketched data $Y_{\text{sketch}\;}\in R^{N\times d}$, sketched reference $X_{\text{sketch}\;}\in R^{K\times d}$		
**Require:** Graph Laplacian $L\in R^{N\times N}$, regularization parameters λ,ρ, tolerance *τ* = 10^−4^		
**Ensure:** Cell type abundances $\beta \in R^{N\times K}$		
1:	// Precomputation	
2:	$G\leftarrow X_{\text{sketch}\;}X_{\text{sketch}\;}^{T}\;$	▷ Gram matrix $\in R^{K\times K}$
3:	Extract neighbor indices 𝒩(i) from L for all spots i	
4:	// Initialization	
5:	$\beta \leftarrow 0_{N\times K}$	▷ Initialize with zeros
6:	t←0	▷ Iteration counter
7:	// Iterative Optimization	
8:	**repeat**	
9:	$\beta^{\text{old}\;}\leftarrow \beta$	
10:	**for** i=1 to N **do**	▷ Iterate over all spots
11:	${𝒩}_{i}\leftarrow 𝒩(i)$	▷ Neighbors of spot i
12:	$d_{i}\leftarrow |𝒩|$	▷ Degree of spot i
13:	**for** k=1 to K **do**	▷ Iterate over all cell types
14:	// Compute partial residual	
15:	$r_{ik}\leftarrow {\left(X_{\text{sketch}\;}Y_{\text{sketch}\;}^{T}\right)}_{ki}$	▷ Data fit term
16:	$r_{ik}\leftarrow r_{ik}-\sum_{j\neq k}\beta_{ij}\cdot G_{jk}$	▷ Subtract other cell types
17:	$r_{ik}\leftarrow r_{ik}+\lambda \sum_{n\in {𝒩}_{i}}\beta_{nk}$	▷ Spatial smoothing term
18:	// Soft-thresholding with non-negativity	
19:	$s\leftarrow sign\left(r_{ik}\right)\cdot max\left(\left|r_{ik}\right|-\rho ,0\right)$	▷ ${𝒮}_{\rho }\left(r_{ik}\right)$
20:	$\beta_{ik}\leftarrow max\left(0,\frac{s}{G_{kk}+\lambda d_{i}}\right)$	▷ Update with projection
21:	**end for**	
22:	**end for**	
23:	// Check convergence	
24:	$\delta \leftarrow {\left\|\beta -\beta^{\text{old}\;}\right\|}_{F}/{\left\|\beta^{\text{old}\;}\right\|}_{F}$	▷ Relative change
25:	t←t+1	
26:	**until** δ<τ or $t\geq t_{max}$	▷ Converged or max iterations
27:	**return** β	

To ensure strictly comparable evaluation, performance metrics for competing methods (Cell2Location, RCTD, Stereoscope, etc.) were obtained directly from the official Spotless benchmark results [11]. FlashDeconv was evaluated on identical source datasets using the same ground-truth labels and metric computation procedures.

Performance metrics include Root Mean Square Error (RMSE), Pearson correlation coefficient, and Area Under the Precision-Recall Curve (AUPR) for rare cell type detection. For Gold Standard data, we additionally report Jensen-Shannon Divergence (JSD) following the Spotless protocol. All runtime and memory benchmarks were performed on an Apple MacBook Pro with M2 Max chip (32GB unified memory) running macOS, representing consumer-grade hardware without GPU acceleration.

## Supplementary Material

Supplement 1

## Data Availability

The Spotless benchmark datasets are available at Zenodo (https://zenodo.org/records/10277187) with code at https://github.com/saeyslab/spotless-benchmark. The Mouse Brain Visium dataset was obtained from the cell2location data portal (https://cell2location.cog.sanger.ac.uk/tutorial/); raw data are available at ArrayExpress (accession E-MTAB-11114 for Visium data, E-MTAB-11115 for snRNA-seq reference) [4]. The Visium HD Mouse Small Intestine (FFPE) dataset was obtained from 10x Genomics (https://www.10xgenomics.com/datasets/visium-hd-cytassist-gene-expression-libraries-of-mouse-intestine). The Xenium Fresh Frozen Mouse Colon dataset, used for ground truth validation of deconvolution accuracy with single-cell resolution data, was obtained from 10x Genomics (https://www.10xgenomics.com/datasets/fresh-frozen-mouse-colon-with-xenium-multimodal-cell-segmentation-1-standard). The intestinal scRNA-seq reference was obtained from Haber et al. [37]; we used the pre-processed version from Zenodo (https://zenodo.org/records/4447233), which contains 10,896 cells with cell type annotations. The original data is deposited at GEO (GSE92332). The human ovarian cancer Visium dataset is available at GEO (https://www.ncbi.nlm.nih.gov/geo/query/acc.cgi?acc=GSE211956) [36].

## Code Availability

FlashDeconv is implemented as an open-source Python package available at https://github.com/cafferychen777/flashdeconv. Complete analysis scripts for reproducing all figures and benchmarks presented in this paper are available at https://github.com/cafferychen777/flashdeconv-reproducibility.
