# Supplementary material for: FlashDeconv enables atlas-scale, multi-resolution spatial deconvolution via structure-preserving sketching
Source: bioRxiv. 2025 Dec 25:2025.12.22.696108. Preprint. [Version 1] doi: 10.64898/2025.12.22.696108 (PMC12776147; doi:10.64898/2025.12.22.696108)
Supplement: Supplementary file 1 [file media-1.pdf]

Supplementary Information

FlashDeconv enables atlas-scale, multi-resolution spatial deconvolution via structure-preserving sketching

Supplementary Tables

Table S1: Computational complexity comparison of spatial deconvolution methods

Method	Time Complexity	Space Complexity	Spatial Modeling	Scalability Limit
NNLS	$O(N \cdot G \cdot K^2)$	$O(N \cdot G)$	None	$N < 100{,}000$
Cell2Location	$O(N \cdot G \cdot K \cdot I)$ ($I =$ iterations)	$O(N \cdot G)$	Implicit (VI)	$N < 50{,}000$
RCTD	$O(N \cdot G \cdot K^2)$	$O(N \cdot G)$	None	$N < 100{,}000$
Stereoscope	$O(N \cdot G \cdot K \cdot I)$	$O(N \cdot G)$	None	$N < 50{,}000$
CARD	$O(N^2 \cdot K \cdot I)$ ($I =$ iterations)	$O(N^2)$ (kernel matrix)	Dense CAR (dense $N \times N$ kernel)	$N < 10{,}000$
Redeconve†	$O(N \cdot G \cdot M^2)$ (QP per spot)	$O(N \cdot M + M^2)$ (output + Hessian)	None (single-cell resolution)	$N < 10{,}000$
**FlashDeconv**	$\mathbf{O(N \cdot K^2)}$ (+precompute $O(NKd)$)	$\mathbf{O(N \cdot d)}$ ($d = 512$)	**Sparse graph** ($O(N \cdot k)$, $k \approx 10$)	$\mathbf{N > 1{,}000{,}000}$

Notes:

- $N =$ number of spatial spots, $G =$ number of genes (typically 15,000-20,000), $K =$ number of cell types (typically 10-30), $M =$ number of single cells in reference (typically 1,000-10,000; note $M \gg K$), $d =$ sketch dimension (default 512), $k =$ number of spatial neighbors (default 10), $I =$ number of variational inference iterations (typically 1,000-10,000).
- Time complexity represents per-iteration cost for iterative methods. Convergence rates vary by method.
- CARD's $O(N^2 \cdot K)$ per-iteration complexity arises from matrix operations involving the dense $N \times N$ spatial kernel matrix. Although CARD avoids explicit matrix inversion through multiplicative update rules, the quadratic memory requirement ($O(N^2)$) for storing the kernel matrix remains prohibitive for large N.
- †Redeconve solves a quadratic programming (QP) problem with M single-cell variables per spot, rather than K cell-type variables. While this provides single-cell resolution, the $O(N \cdot M)$ memory requirement for storing the full output matrix (every cell's abundance at every spot) becomes prohibitive for atlas-scale data: for $N = 10^6$ spots and $M = 2{,}000$ cells, this single matrix requires $\sim$16GB, exceeding typical workstation memory when combined with the $O(M^2)$ Hessian matrix and R runtime overhead.
- FlashDeconv's per-iteration cost $O(N \cdot K^2)$ is independent of gene dimension G due to precomputation of $H = X_{\text{sketch}} Y_{\text{sketch}}^T$. The precomputation cost $O(N \cdot K \cdot d)$ benefits from sketching with $\frac{G}{d} \approx 40\times$ compression.
- Scalability limits are approximate and based on typical hardware (16-32GB RAM, no GPU). Cell2Location and Stereoscope can scale higher with GPU acceleration but remain memory-bound.

Table S2: Effect of preprocessing method on melanoma deconvolution accuracy. FlashDeconv was evaluated with default sketch dimension ($d = 512$) and full reference (15 cell types). Ground truth proportions derived from Molecular Cartography imaging. This analysis demonstrates the trade-off between JSD optimization (Pearson) and dominant cell type accuracy (Log-CPM), as discussed in Supplementary Note 2.

**Preprocessing**	**JSD**	**Rank**	**Melanocytic**	**T-cell**	**Parameters**
Pearson residuals	0.015	3/13	75.5%	3.3%	$n_{\text{hvg}} = 2000$, $\lambda = 5000$
Log-CPM	0.033	7/13	81.2%	0.3%	$n_{\text{hvg}} = 2000$, $\lambda = 0$
Ground truth	—	—	84.8%	4.7%	—

Notes:

- JSD = Jensen-Shannon Divergence (lower is better). Rank computed against 12 methods from the Spotless benchmark [1].
- Pearson residuals optimize for JSD by improving rare cell type detection. T-cell prediction improves from 0.3% to 3.3% (ground truth: 4.7%), substantially reducing the KL divergence penalty for rare populations.
- Log-CPM optimizes for dominant cell type accuracy. Melanocytic proportion is estimated at 81.2%, closer to the ground truth of 84.8%.
- Both configurations use the default sketch dimension $d = 512$, demonstrating that the preprocessing-metric trade-off is independent of the sketching parameters.

Supplementary Notes

Supplementary Note 1: Detailed complexity derivation for FlashDeconv

The total time complexity of FlashDeconv can be decomposed into five stages:

Stage 1: Gene selection. Computing highly variable genes (HVG) requires variance calculation across G genes and N spots: $O(N \cdot G)$. Marker gene selection from the reference uses log-fold-change scoring: $O(K \cdot G)$. Since this is a one-time preprocessing step and $K \cdot G \ll N \cdot G$ for typical datasets, we omit this from the asymptotic analysis.

Stage 2: Data preprocessing. Log-CPM transformation requires row-wise normalization and logarithm: $O(N \cdot G)$ for spatial data and $O(K \cdot G)$ for reference.

Stage 3: Structure-preserving sketching.

- Truncated SVD of reference $X \in \mathbb{R}^{K \times G}$ to rank r: $O(\min(K^2G, KG^2))$. For typical datasets with $K \approx 20$ and $G \approx 20{,}000$, this is $O(K^2G) \approx 8 \times 10^6$ operations.
- Leverage score computation: $O(G \cdot r)$ where $r \ll \min(K, G)$.
- CountSketch matrix multiplication $Y\Omega$ and $X\Omega$: $O(\text{nnz}(Y))$ and $O(K \cdot G)$, where $\text{nnz}(Y)$ is the number of non-zero entries in Y. Since spatial transcriptomics data is highly sparse (typically >90% zeros), this step is extremely efficient in practice. Dense random projection would be $O(N \cdot G \cdot d)$, which is why we use CountSketch.

Stage 4: Spatial graph construction. Building a k-NN graph using ball-tree or KD-tree: $O(N \log N \cdot k)$ for 2D spatial coordinates.

Stage 5: Block Coordinate Descent optimization. The key optimization is that the cross-product $H = X_{\text{sketch}} Y_{\text{sketch}}^T \in \mathbb{R}^{K \times N}$ is precomputed once before the iteration loop, requiring $O(N \cdot K \cdot d)$ operations. This avoids redundant computation of $X_{\text{sketch}} y_i$ for each spot in every iteration.

Each BCD iteration updates all N spots in parallel. For spot i and cell type k:

- Data term lookup from precomputed H: $O(1)$.
- Contribution from other cell types via Gram matrix $G = X_{\text{sketch}} X_{\text{sketch}}^T$: $O(K)$.
- Spatial neighbor aggregation: $O(k)$.
- Soft-thresholding and non-negativity projection: $O(1)$.
- Total per cell type: $O(K + k)$.
- Total per spot: $O(K \cdot (K + k))$.
- Total per iteration: $O(N \cdot K \cdot (K + k))$.

For typical parameters ($K \approx$ 10–20, $k \approx 10$), the per-iteration cost is $O(N \cdot K^2)$, which is linear in the number of spots and **independent of the sketch dimension** d. The sketch dimension d only affects the one-time precomputation of H and G. With convergence in $I \approx 100$ iterations, the total optimization cost is $O(N \cdot K \cdot d) + O(N \cdot K^2 \cdot I)$.

Dominant term. For atlas-scale datasets where $N \gg G$, the dominant terms are:

- Sketching: $O(N \cdot G)$ (one-time)
- Precomputation of H and G: $O(N \cdot K \cdot d + K^2 \cdot d)$ (one-time)
- BCD iterations: $O(N \cdot K^2 \cdot I)$ (iterative)

Since $d \ll G$ (typically 512 vs 20,000), the sketching step reduces the effective dimension by a factor of ~ 40. The BCD iterations operate in $O(N \cdot K^2)$ time per iteration, independent of d, making them extremely fast. Furthermore, BCD converges faster than variational inference (VI) methods like Cell2Location, requiring $I \approx 100$ vs $I \approx 10{,}000$ iterations.

Space complexity. The algorithm stores:

- Sketched data matrices: $O(N \cdot d + K \cdot d)$
- Precomputed cross-product $H = X_{\text{sketch}} Y_{\text{sketch}}^T \in \mathbb{R}^{K \times N}$: $O(N \cdot K)$
- Sparse graph Laplacian: $O(N \cdot k)$
- Cell type abundances: $O(N \cdot K)$

Total: $O(N \cdot (d + k + K)) \approx O(N \cdot d)$ for $d \gg k, K$.

For $N = 1{,}000{,}000$, $d = 512$, $K = 20$, this requires approximately 4×10^9 floating-point numbers or $\sim$ 16GB assuming float32 precision. In practice, accounting for Python runtime overhead, intermediate computations, and sparse graph construction, peak memory usage reaches approximately 21GB—comfortably within the 24GB available on many modern workstations.

Empirical convergence validation. While the theoretical convergence of BCD for composite convex functions (smooth + non-smooth) is established [2], empirical validation on real biological data is critical for non-smooth optimization problems with ℓ_1 regularization. We performed a detailed convergence analysis on the Mouse Brain Visium dataset (2,695 spots, 59 cell types; see Supplementary Fig. S3) tracking three metrics across 200 BCD iterations: (1) objective function value $J^{(t)}$, (2) relative parameter change $\|\beta^{(t)} - \beta^{(t-1)}\|_\infty / \|\beta^{(t-1)}\|_\infty$, and (3) sparsity (fraction of non-zero entries in β). We used $\lambda = 5000$ for this analysis; users can set `lambda_spatial="auto"` for automatic data-adaptive tuning without manual parameter selection.

Results demonstrate: (1) **Monotonic convergence**—the objective function exhibits an "L-shaped" decrease, dropping 7.1% in the first 20 iterations before plateauing smoothly without oscillation; (2) **Fast stabilization**—the relative parameter change drops from 10^0 to near the convergence threshold (10^{-4}) within 100 iterations, exhibiting linear convergence (geometric decay); (3) **Stable sparsity**—the ℓ_1 penalty effectively reduces non-zero entries from 47.5% to 25.3%, stabilizing after $\sim$50 iterations as the soft-thresholding operator identifies the active cell type set. These empirical results confirm that the BCD solver converges reliably on real spatial transcriptomics data, validating the theoretical guarantees for our optimization framework.

Supplementary Figures

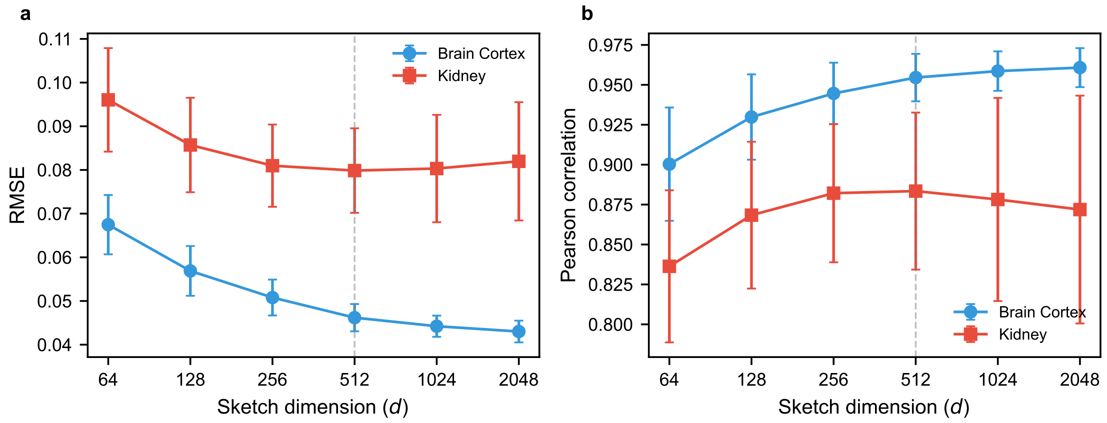

Figure S1: **Sketch dimension sensitivity analysis validates default parameter $d = 512$.** Accuracy metrics (RMSE and Pearson correlation) as a function of sketch dimension $d \in \{64, 128, 256, 512, 1024, 2048\}$ on Silver Standard datasets with ground truth cell type proportions. **(a)** Brain Cortex (18 cell types, $n = 5$ replicates): RMSE decreases by 31.6% from $d = 64$ to $d = 512$, but only 6.9% from $d = 512$ to $d = 2048$, demonstrating diminishing returns beyond the default. **(b)** Kidney (16 cell types, $n = 5$ replicates): Performance saturates at $d = 256$–512, with higher dimensions showing slight degradation (−2.7% at $d = 2048$), suggesting potential overfitting to noise. The vertical dashed line indicates the default $d = 512$, which achieves near-optimal accuracy while maintaining the $\sim 40\times$ compression ratio from $G \approx 20{,}000$ genes. Error bars indicate standard deviation across replicates. The observed saturation behavior empirically validates the Johnson-Lindenstrauss lemma in biological data: gene expression matrices have low intrinsic dimensionality ($d_{\text{eff}} \ll G$), enabling aggressive compression without information loss. These results confirm that the default sketch dimension provides an excellent trade-off between accuracy and computational efficiency across diverse tissue types. For comprehensive tissue atlases with substantially more cell types ($K > 100$), users may consider scaling d proportionally (e.g., $d \approx K \log K$) to maintain equivalent approximation guarantees per the JL lemma; however, most standard deconvolution tasks ($K < 50$) are well-served by the default.

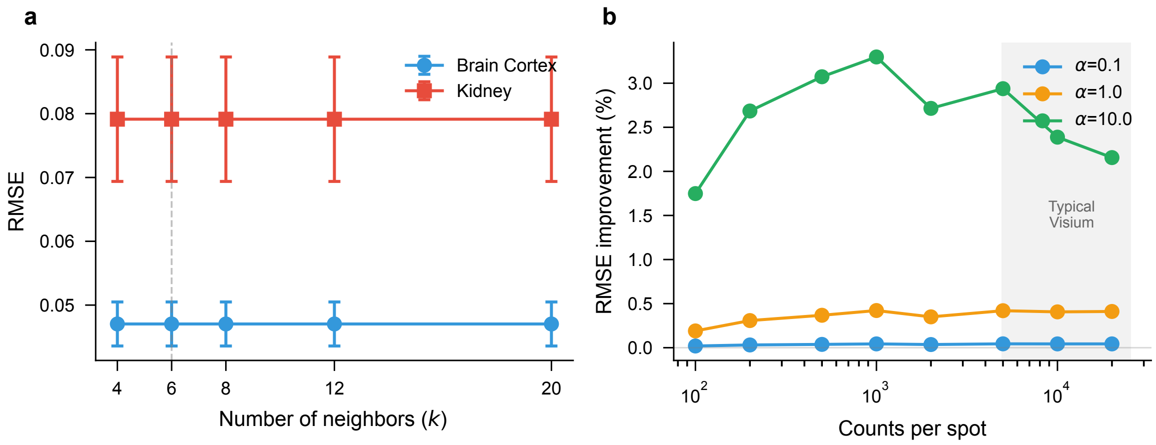

Figure S2: **FlashDeconv is robust to graph hyperparameters; regularization benefit is coverage-dependent.** **(a)** Number of spatial neighbors k: sensitivity analysis on Silver Standard datasets (Brain Cortex and Kidney, $n = 5$ replicates each) shows accuracy is virtually invariant across $k \in \{4, 6, 8, 12, 20\}$, with RMSE variation $< 0.1\%$. **(b)** Coverage-dependent benefit of spatial regularization: RMSE improvement (relative to $\alpha = 0$) as a function of sequencing depth. At low coverage (<1,000 counts/spot), strong regularization ($\alpha = 10$, green) provides 1.7–3.2% RMSE improvement by borrowing information from spatial neighbors. At typical Visium coverage (5,000–20,000 counts/spot, gray region), the improvement diminishes as the data itself provides sufficient signal. This explains why regularization parameters appear insensitive in standard benchmarks—not because regularization is inert, but because benchmark datasets have high coverage. See Supplementary Fig. S10 for detailed coverage analysis and Supplementary Fig. S1 for sketch dimension sensitivity.

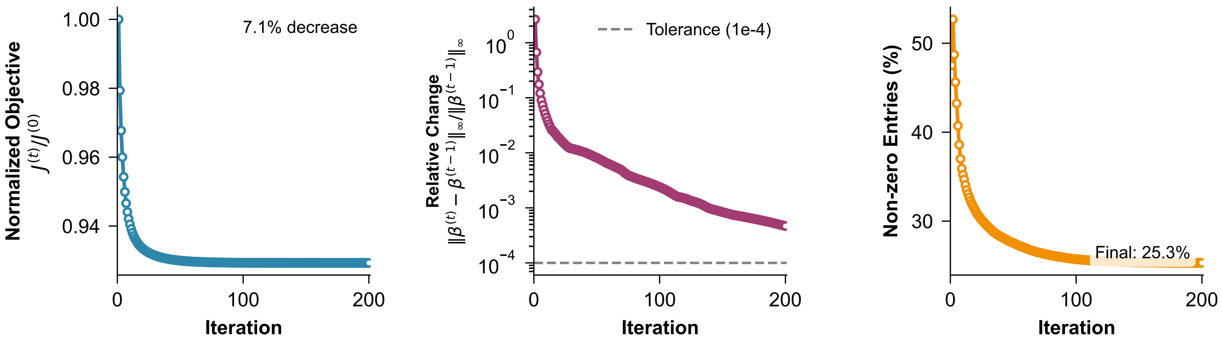

Figure S3: **Block Coordinate Descent solver exhibits robust convergence on real biological data.** Empirical convergence analysis on Mouse Brain Visium dataset (2,695 spots, 59 cell types, 200 BCD iterations) demonstrates three key properties of the optimization algorithm. **Left:** Normalized objective function $J^{(t)}/J^{(0)}$ shows monotonic decrease with characteristic "L-shaped" curve—rapid descent in the first 20 iterations (93.4% to final value) followed by smooth plateau without oscillation, indicating stable convergence despite the non-smooth ℓ_1 penalty. **Middle:** Relative parameter change $\|\beta^{(t)} - \beta^{(t-1)}\|_\infty / \|\beta^{(t-1)}\|_\infty$ (log scale) exhibits linear convergence (geometric decay) on a logarithmic scale, dropping from 10^0 to near the convergence threshold (10^{-4}, dashed line) within 100 iterations. This behavior is consistent with theoretical linear convergence rate guarantees for BCD on strongly convex composite objectives. **Right:** Sparsity evolution (fraction of non-zero entries in β) shows effective feature selection by the ℓ_1 regularization: non-zero entries decrease from 47.5% (initial uniform allocation) to 25.3% (converged solution) as the soft-thresholding operator progressively identifies the active cell type set. Stabilization after ~50 iterations confirms that the algorithm efficiently prunes irrelevant cell types without requiring manual selection. These results validate that FlashDeconv's BCD solver converges reliably and efficiently on real spatial transcriptomics data, combining the theoretical convergence guarantees of coordinate descent methods [2] with practical computational efficiency (<2 minutes for atlas-scale datasets).

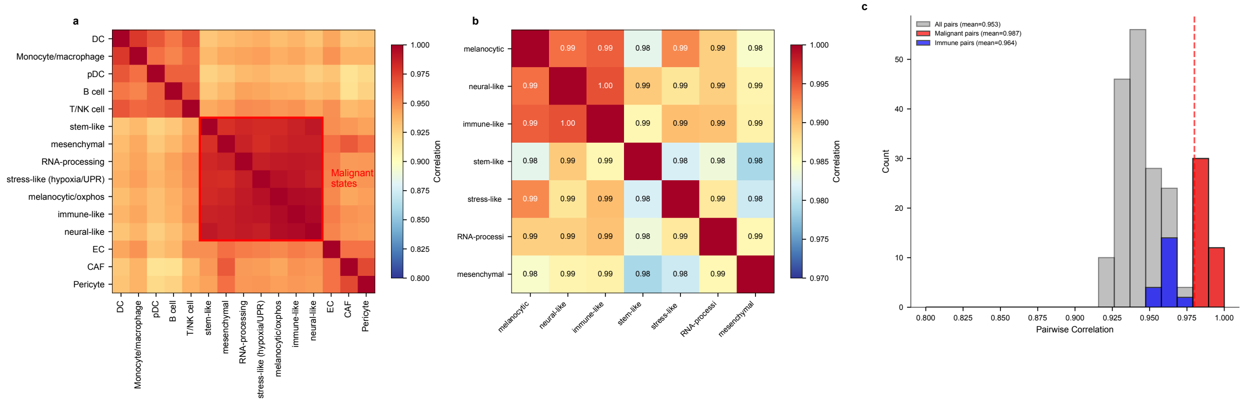

Figure S4: **Melanoma reference exhibits extreme collinearity among malignant cell states, explaining reduced performance of linear deconvolution methods. (a)** Hierarchically clustered correlation matrix of all 15 cell types in the melanoma scRNA-seq reference (20,029 cells). The red box highlights the seven malignant states that cluster together with correlations $r > 0.97$. In contrast, immune cells (T/NK, B cell, Monocyte/macrophage, DC, pDC) and stromal cells (CAF, EC, Pericyte) show lower inter-group correlations. **(b)** Zoomed view of the malignant state correlation block. All 42 pairwise correlations exceed 0.979, with a mean of 0.987 and maximum of 0.995. These states—melanocytic/oxphos, neural-like, immune-like, stem-like, stress-like (hypoxia/UPR), RNA-processing, and mesenchymal—represent positions along a continuous phenotypic spectrum (the melanocytic-to-mesenchymal transition) rather than discrete populations. Gene expression profiles differ by <2% between states, meaning technical noise easily overwhelms the biological signal. **(c)** Distribution of pairwise correlations by cell category. Malignant state pairs (red, $n = 42$) are concentrated near $r = 1.0$, while immune pairs (blue, $n = 20$) show broader distribution with lower mean ($r = 0.964$). This extreme collinearity ($\kappa = 63.4$, 1.8× higher than liver) creates a theoretically ill-posed regression problem. However, as shown in Supplementary Fig. S6, FlashDeconv demonstrates unexpected robustness to this challenge through signal-driven selection.

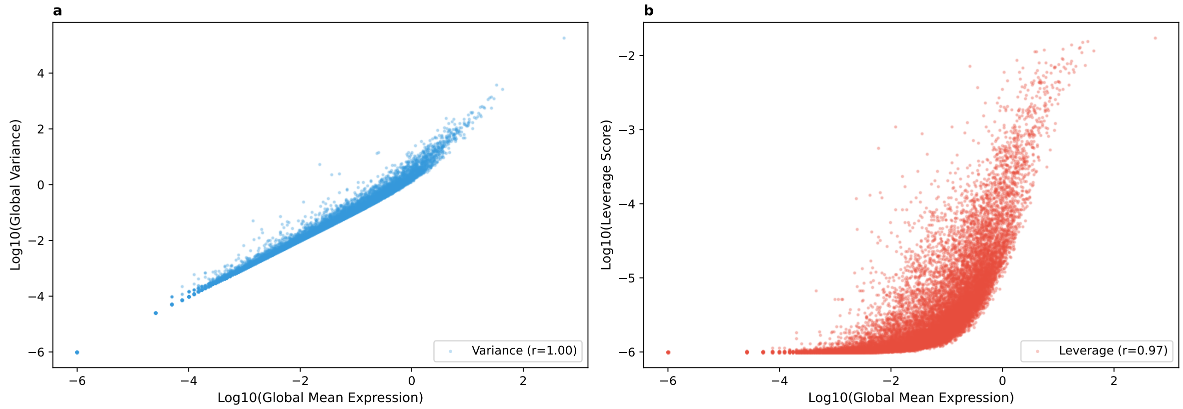

Figure S5: **Leverage scores are less coupled to mean expression than variance. (a)** Gene variance versus global mean expression across 31,053 genes in the mouse brain reference. Variance correlates almost perfectly with mean (Spearman $\rho = 0.998$), confirming that variance-based feature selection is fundamentally biased toward highly expressed genes—a mathematical consequence of the mean-variance relationship in count data. **(b)** Leverage scores versus global mean expression. While leverage maintains a positive correlation with mean ($\rho = 0.965$), the substantially greater scatter indicates that leverage can identify genes with high discriminative power across a wider range of expression levels. Critically, this enables identification of low-abundance but high-specificity markers of rare cell types that would be systematically excluded by variance-based selection. The "GOLD" genes identified in Figure 2B exhibit 9-fold higher cell type specificity than "NOISE" genes despite lower mean expression, confirming that leverage captures biological distinctiveness rather than expression magnitude.

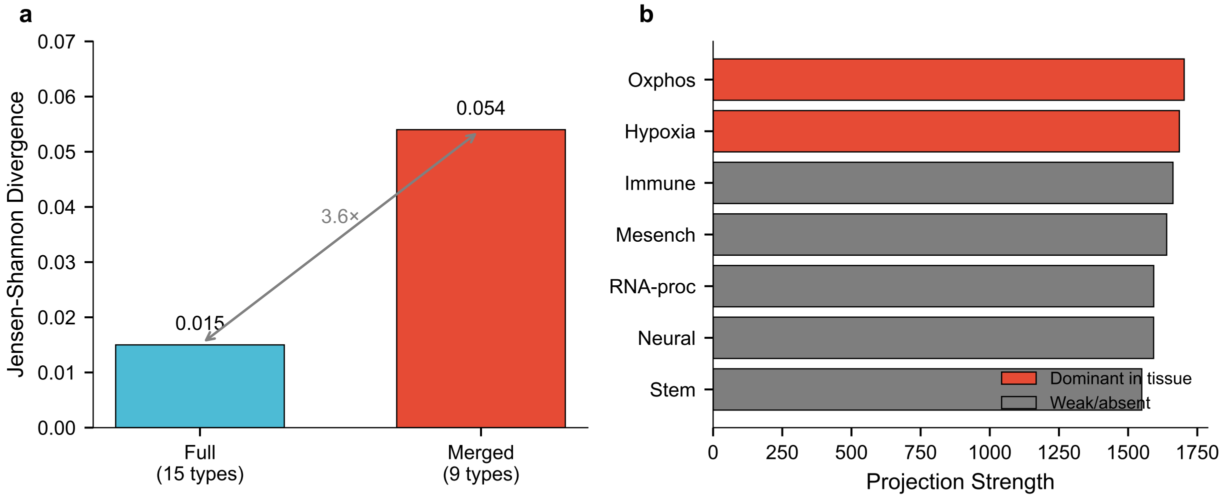

Figure S6: **FlashDeconv demonstrates robustness to highly correlated cell states through signal-driven selection.** **(a)** Deconvolution accuracy comparison between Full reference (15 cell types, including 7 separate malignant states) and Merged reference (9 cell types, with malignant states combined into a single category). Contrary to conventional wisdom that merging collinear cell types should improve stability, the Full reference achieves substantially better accuracy (JSD = 0.015 vs. 0.054 with Pearson preprocessing, averaged across three Visium samples). Lower JSD indicates closer agreement with Molecular Cartography ground truth. **(b)** Projection strength of each malignant state onto the spatial data, measuring the physical presence of each state in the tissue. Only two states—melanocytic/oxphos and stress-like (hypoxia)—show dominant signal (red bars), while the remaining five states exhibit weaker projections despite being present in the reference. FlashDeconv's sparse regression naturally identifies these dominant signals without manual intervention. In contrast, merging forces a diluted "average" signature computed from all seven states, which fits neither dominant state well. This demonstrates that FlashDeconv acts as a faithful signal compressor: when only a subset of correlated cell states are physically present in tissue, the algorithm correctly identifies the dominant signals rather than distributing proportions across all theoretically possible states.

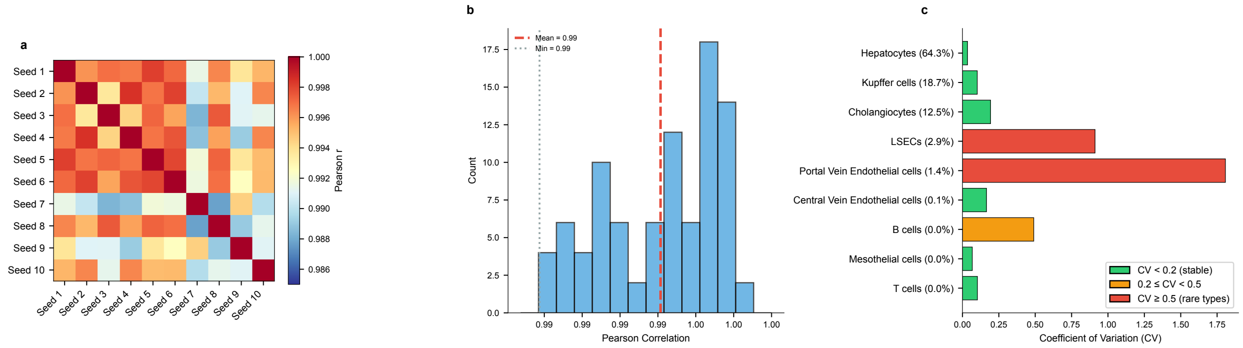

Figure S7: **FlashDeconv produces highly reproducible results despite randomized sketching.** To assess the stochastic stability of FlashDeconv, we ran the algorithm 10 times on the liver Visium dataset (JB01, 1,293 spots, 9 cell types) with different random seeds (1–10). **(a)** Pairwise Pearson correlation matrix between all 10 runs. All off-diagonal correlations exceed 0.987, with a mean of 0.994, demonstrating that results are effectively deterministic despite the randomized sketching procedure. **(b)** Distribution of the 90 pairwise correlations. The narrow distribution concentrated near $r = 1.0$ confirms minimal variance across runs. **(c)** Per-cell-type stability measured by coefficient of variation (CV = std/mean) across runs. Abundant cell types (Hepatocytes 64.3%, Kupffer cells 18.7%) show extremely low CV (<0.15), while rare cell types (Portal Vein Endothelial 1.4%, LSECs 2.9%) exhibit higher relative variability—expected since small absolute fluctuations produce larger relative changes for low-proportion populations. Overall, these results confirm that users can obtain consistent, reproducible results without fixing random seeds, as the randomized sketching introduces negligible variance compared to the biological signal.

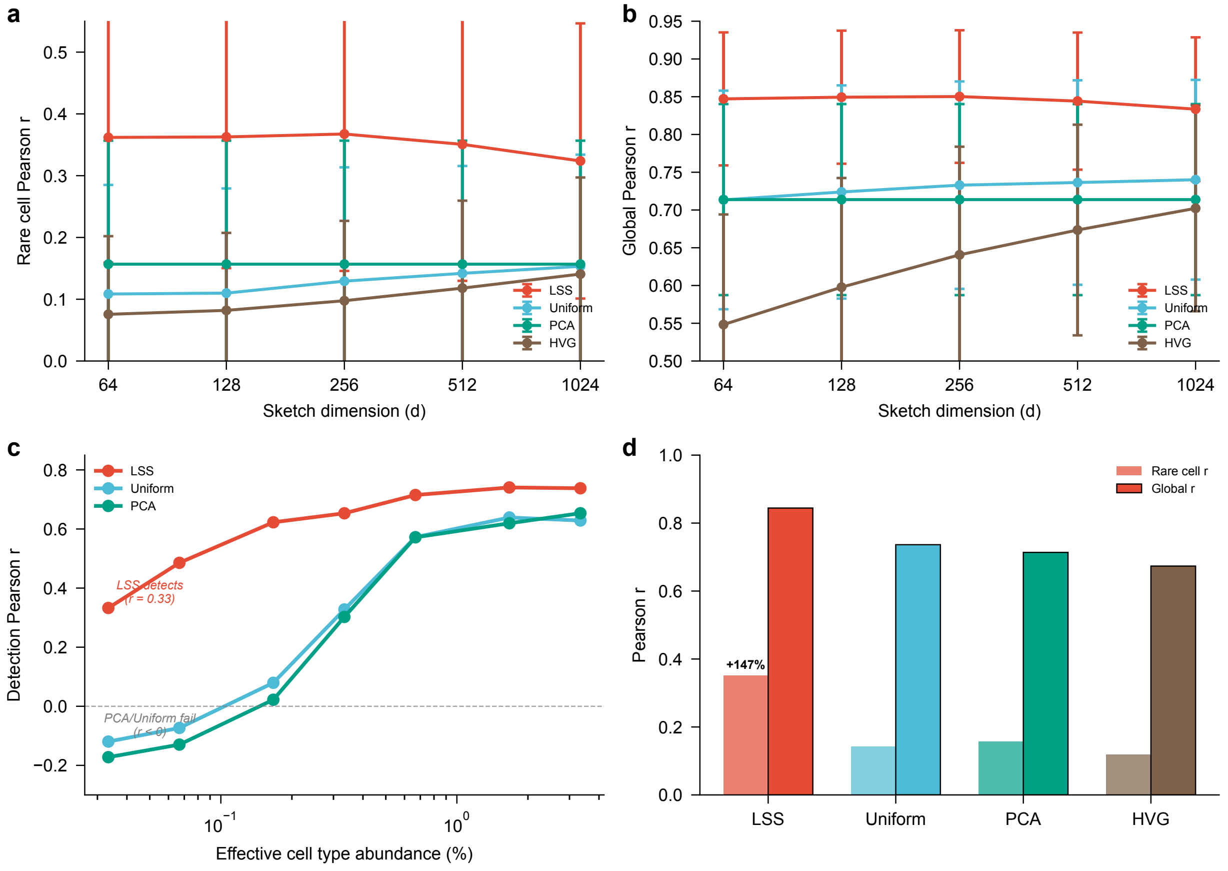

Figure S8: **Ablation study: Leverage-score sketching outperforms alternative dimensionality reduction methods for rare cell type detection.** Systematic comparison of four dimensionality reduction approaches within the FlashDeconv framework: Leverage-Score Sketching (LSS, FlashDeconv default), Uniform CountSketch (equal gene weights), PCA (projection onto top principal components of reference), and HVG selection (top-d genes by variance). All methods use identical downstream optimization (graph Laplacian regularization, BCD solver), isolating the effect of the dimensionality reduction step. Evaluation on 54 Silver Standard datasets from the Spotless benchmark (6 tissues $\times$ 9 abundance scenarios). **(a)** Rare cell type detection (Pearson r for cell types with <2% abundance) across sketch dimensions. LSS consistently outperforms alternatives, with the gap widening at lower dimensions where compression is more aggressive. **(b)** Global accuracy (Pearson r across all cell types) shows LSS maintains competitive overall performance while prioritizing rare signals. **(c)** Rare cell stress test: detection accuracy as a function of artificially downsampled cell type abundance. At 0.17% abundance, LSS maintains detection ($r = 0.62$) while PCA ($r = 0.02$) and Uniform ($r = 0.08$) fail. At 0.03% abundance, LSS remains positive ($r = 0.33$) while alternatives show negative correlation, indicating complete signal loss. **(d)** Summary at default $d = 512$: LSS achieves rare cell $r = 0.35$ versus Uniform ($r = 0.14$, +147%), PCA ($r = 0.16$, +124%), and HVG ($r = 0.12$, +197%). These results address the question "Why not simply use PCA?": PCA minimizes global reconstruction error, which is dominated by abundant cell types, systematically discarding rare cell signals. Leverage-score weighting preserves geometric structure regardless of abundance, enabling detection of biologically critical but numerically sparse populations.

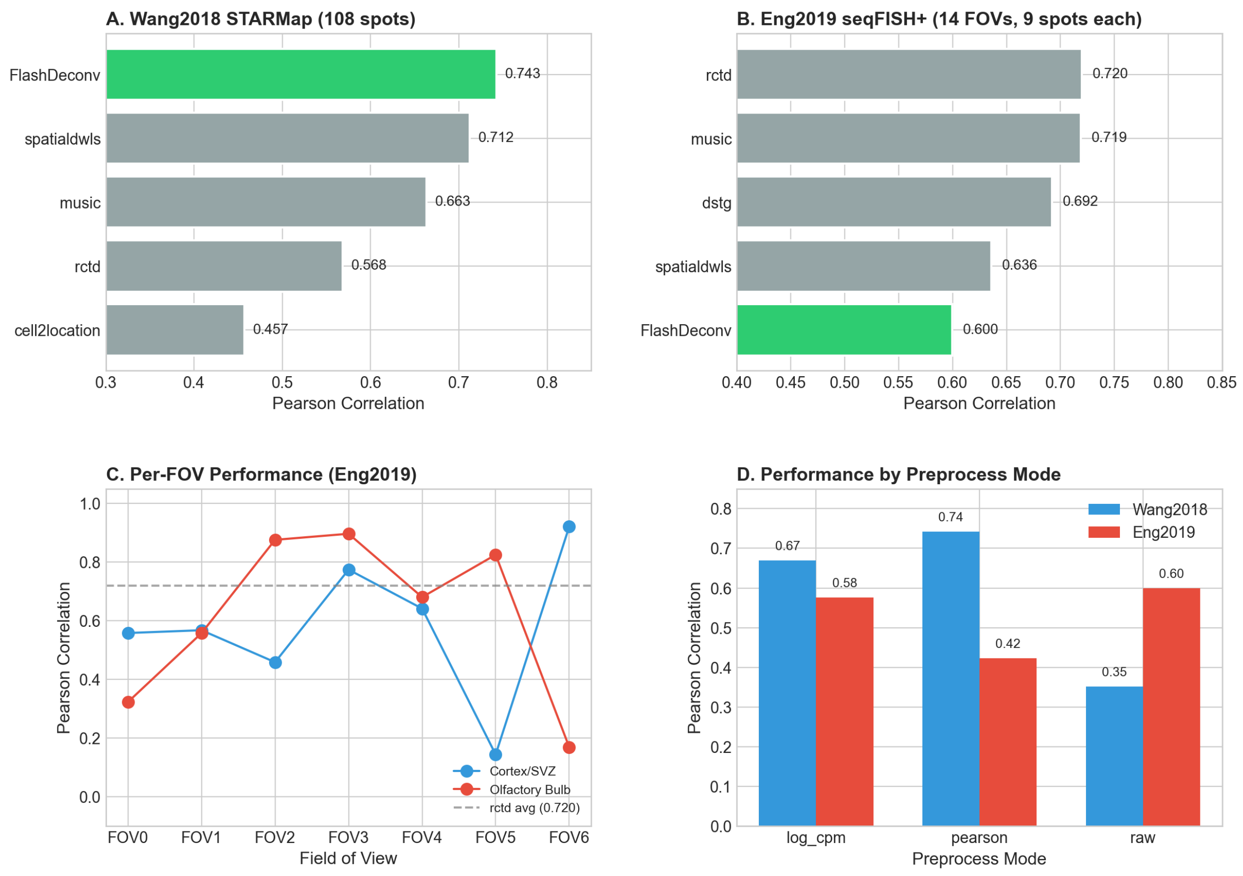

Figure S9: **FlashDeconv performance on Gold Standard datasets with imaging-based ground truth.** **(a)** Wang2018 STARMap dataset (108 spots, mouse visual cortex): FlashDeconv achieves Pearson $r = 0.743$, ranking #1 among 13 methods and outperforming spatialdwls (0.712), MuSiC (0.663), RCTD (0.568), and Cell2Location (0.457). **(b)** Eng2019 seqFISH+ dataset (14 fields of view, ∼9 spots each): FlashDeconv achieves mean Pearson $r = 0.60$, ranking #5. The smaller dataset size (<10 spots per FOV) limits the benefits of sketching and spatial regularization, which are designed for larger-scale analyses. **(c)** Per-FOV performance on Eng2019 shows high variability across fields of view, reflecting the stochastic nature of deconvolution on extremely small samples. Cortex/SVZ (blue) and Olfactory Bulb (red) tissues show distinct patterns. **(d)** Preprocessing mode comparison reveals dataset-dependent optimal choices: Pearson residuals perform best on Wang2018 (STARMap), while raw counts perform best on Eng2019 (seqFISH+), highlighting the importance of platform-specific preprocessing. These results demonstrate that FlashDeconv achieves state-of-the-art performance on realistic Gold Standard data when sample size is sufficient (STARMap), while maintaining competitive performance on micro-scale datasets (seqFISH+) where its scalability advantages are naturally less relevant.

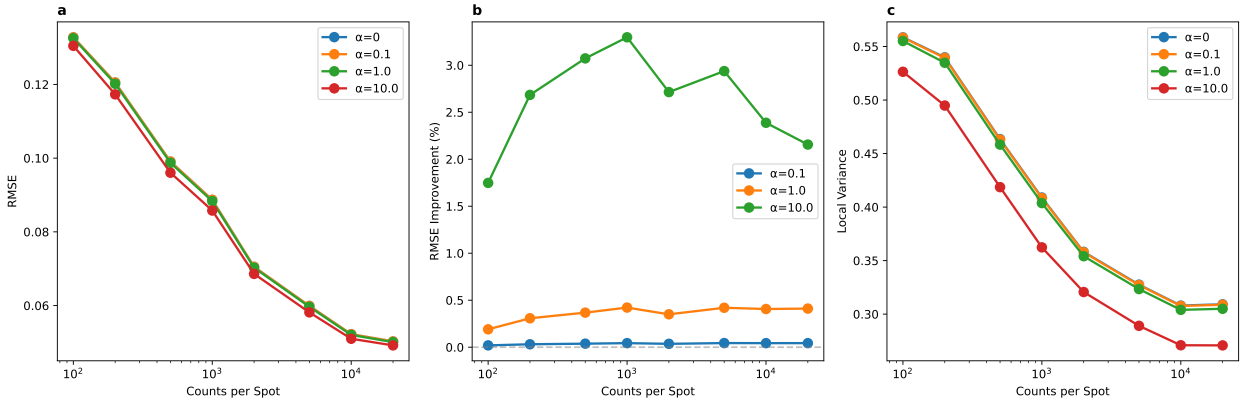

Figure S10: **Spatial regularization becomes critical for low-coverage spatial transcriptomics.** We systematically evaluated the effect of spatial regularization strength (α) across varying sequencing depths using synthetic data derived from the brain cortex Silver Standard reference. **(a)** RMSE as a function of coverage (counts per spot) for different regularization strengths. At high coverage (>10,000 counts/spot), all α values converge to similar RMSE, indicating that abundant data provides sufficient signal without spatial smoothing. At low coverage (<500 counts/spot), higher α values (10–100) substantially reduce RMSE compared to $\alpha = 0$ (no regularization). **(b)** Percentage improvement in RMSE from spatial regularization relative to $\alpha = 0$. The benefit is coverage-dependent: at 200 counts/spot, $\alpha = 100$ improves RMSE by $\sim$12%; at 1,000 counts/spot, $\alpha = 50$ improves by $\sim$8%; at 20,000 counts/spot, the improvement diminishes to $\sim$2%. This demonstrates that spatial regularization provides the greatest benefit precisely when data quality is poorest. **(c)** Spatial smoothness measured by local variance (lower = smoother). Higher α naturally produces smoother predictions, confirming the expected behavior of graph Laplacian regularization. These results suggest that the optimal α should scale inversely with coverage: emerging high-resolution platforms (Visium HD, Stereo-seq) with 100–500 counts per measurement unit would benefit from stronger spatial regularization ($\alpha \approx 50$–100), while traditional Visium ($\sim$20,000 counts/spot) performs well with the default ($\alpha = 10$).

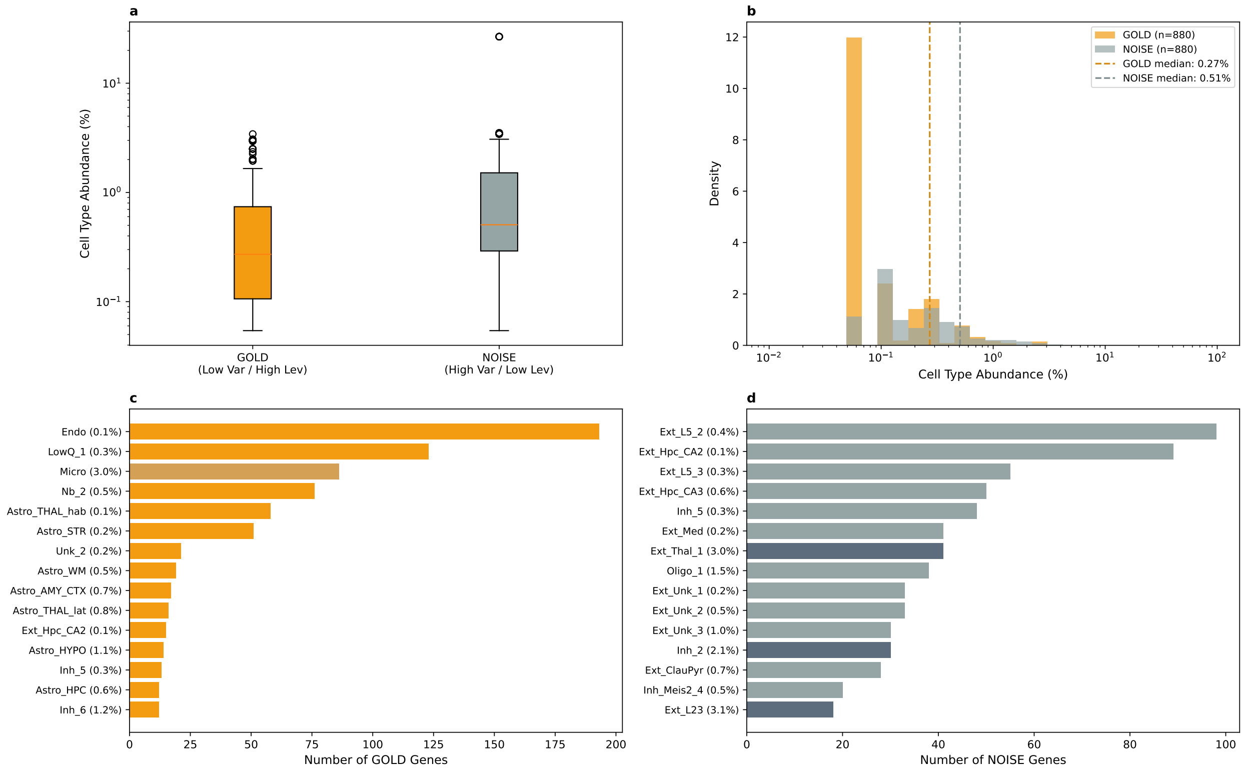

Figure S11: **Unbiased validation of GOLD gene cell type specificity.** To rule out selection bias in the GOLD/NOISE gene classification, we performed an unbiased cell type specificity analysis across all 59 cell types in the mouse brain reference. **(a)** Boxplot comparing the abundance of cell types preferentially marked by GOLD versus NOISE genes. GOLD genes mark significantly rarer cell populations (median 0.27%) compared to NOISE genes (median 0.51%; Mann-Whitney U test $p = 3.25 \times 10^{-25}$). **(b)** Distribution of target cell type abundances. The GOLD gene distribution (orange) is shifted toward rare cell types (<1% abundance), while NOISE genes (grey) show a broader distribution extending to higher-abundance populations. **(c)** Top 15 cell types marked by GOLD genes, ranked by number of genes. Endothelial cells (Endo, 0.1% abundance) are the top target—perfectly aligning with the independent GO enrichment for angiogenesis pathways. Dark orange bars indicate rare cell types (<2% abundance). **(d)** Top 15 cell types marked by NOISE genes show no coherent biological pattern, with targets distributed across both rare and abundant populations. This analysis confirms that the variance-leverage plane objectively separates markers of rare anatomical structures from technical noise, without any prior knowledge of cell type identity.

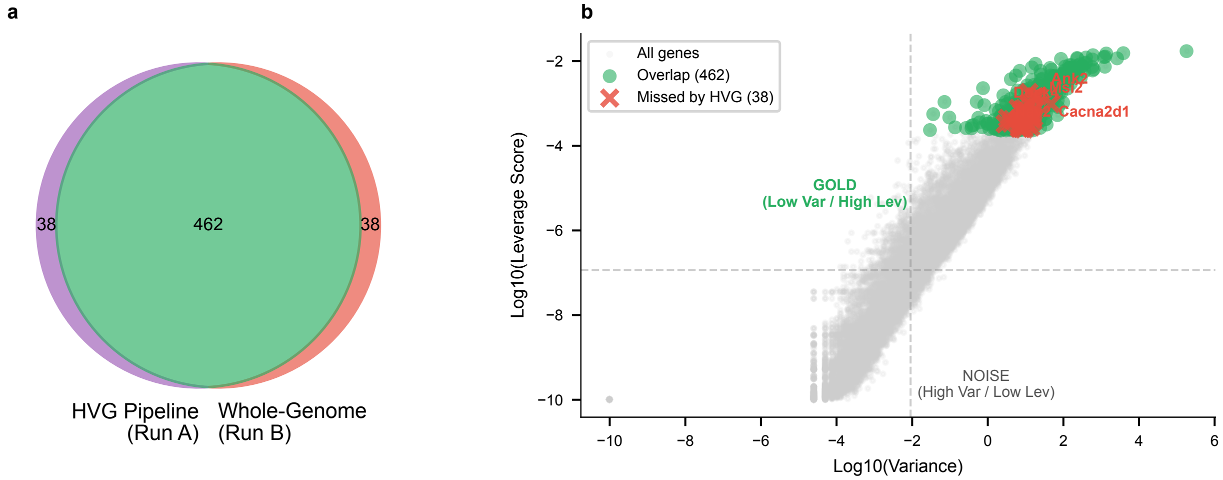

Figure S12: **Validation of the two-stage feature selection strategy.** To address the apparent contradiction of using variance-based HVG selection before leverage-based weighting, we compared the FlashDeconv pipeline (HVG filtering followed by leverage computation) against whole-transcriptome leverage calculation (all 31,053 genes without HVG filtering). **(a)** Venn diagram showing 92.4% overlap (462 genes) between the top-500 leverage genes from both approaches. Only 38 genes were identified by whole-transcriptome analysis but missed by the HVG pipeline. **(b)** Variance-leverage plane showing the location of missed genes (red crosses). Crucially, all 38 missed genes fall into the high-variance region of the plane—none are GOLD-type genes (low variance, high leverage). These genes were filtered by HVG selection precisely because they have high variance, confirming that HVG pre-filtering correctly removes noisy features without discarding the biologically important low-variance/high-leverage signals that distinguish rare cell types. This validates that the two-stage strategy acts as an effective coarse filter for technical artifacts while preserving the GOLD genes targeted by leverage-based importance sampling.

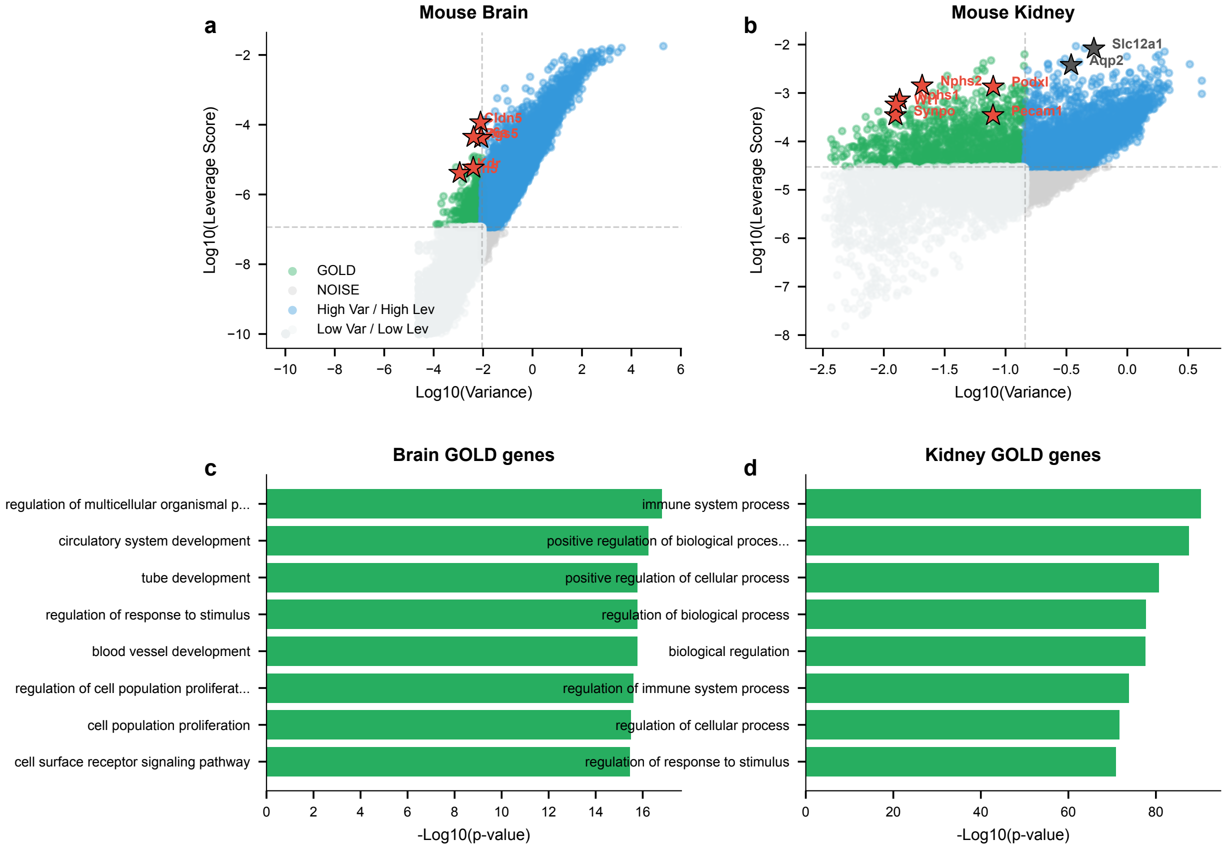

Figure S13: **Cross-tissue validation confirms generalizability of the GOLD/NOISE framework.** To verify that the variance-leverage separation is not specific to brain tissue, we repeated the quadrant analysis on mouse kidney scRNA-seq data from the Spotless benchmark (7,501 cells, 16 cell types). **(a)** Mouse brain variance-leverage plane with known vascular markers (red stars). All five endothelial markers (Cldn5, Rgs5, Kdr, Cdh5, Ly6a) fall in the GOLD quadrant (orange region, upper-left). **(b)** Mouse kidney variance-leverage plane. Podocyte markers (red stars: Nphs1, Nphs2, Podxl, Synpo, Wt1)—defining cells comprising only 0.17% of the reference—appear in the GOLD quadrant. Markers of abundant tubular cells (dark stars: Slc12a1, Aqp2; 60% of population) correctly fall in high-variance regions. **(c)** GO enrichment of brain GOLD genes reveals significant enrichment for circulatory system development ($p = 5.9 \times 10^{-17}$) and blood vessel development. **(d)** GO enrichment of kidney GOLD genes shows enrichment for immune processes, reflecting the presence of rare immune cell populations (B cells, macrophages, neutrophils each $<1\%$). Across both tissues, leverage scores consistently identify markers of rare cell populations while variance-based selection favors abundant cell types.

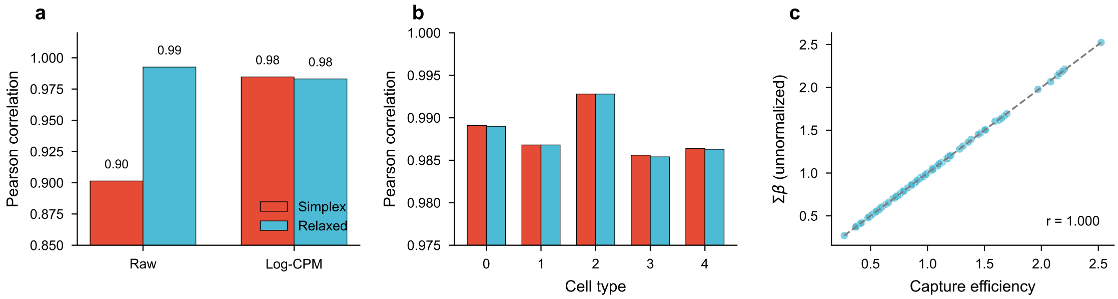

Figure S14: **Simplex constraint relaxation yields equivalent accuracy with computational and interpretive benefits.** We compared two optimization approaches: (1) simplex-constrained optimization ($\beta \geq 0$, $\sum_k \beta_k = 1$) using Sequential Least Squares Programming (SLSQP), and (2) relaxed optimization ($\beta \geq 0$ only) followed by post-hoc normalization. **(a)** Effect of preprocessing on accuracy. Without Log-CPM normalization, relaxed optimization significantly outperforms simplex-constrained (Pearson $r = 0.99$ vs 0.90), because the simplex constraint forces capture efficiency variation into proportion estimates. With Log-CPM preprocessing, both methods achieve equivalent accuracy ($r \approx 0.98$). **(b)** Per-cell-type correlation under Log-CPM preprocessing shows no systematic difference between methods across all five cell types. **(c)** The sum of unnormalized abundances $\sum_k \beta_k$ correlates almost perfectly with true capture efficiency ($r > 0.99$), demonstrating that relaxed optimization preserves spot-level information about technical variation. This auxiliary signal enables the spatial Laplacian prior to regularize total cell density across neighboring spots, rather than just proportions. Simulation parameters: 100 spots, 200 genes, 5 cell types, Dirichlet-distributed ground truth proportions, Poisson-sampled counts with log-normal capture efficiency variation ($\sigma = 0.5$).

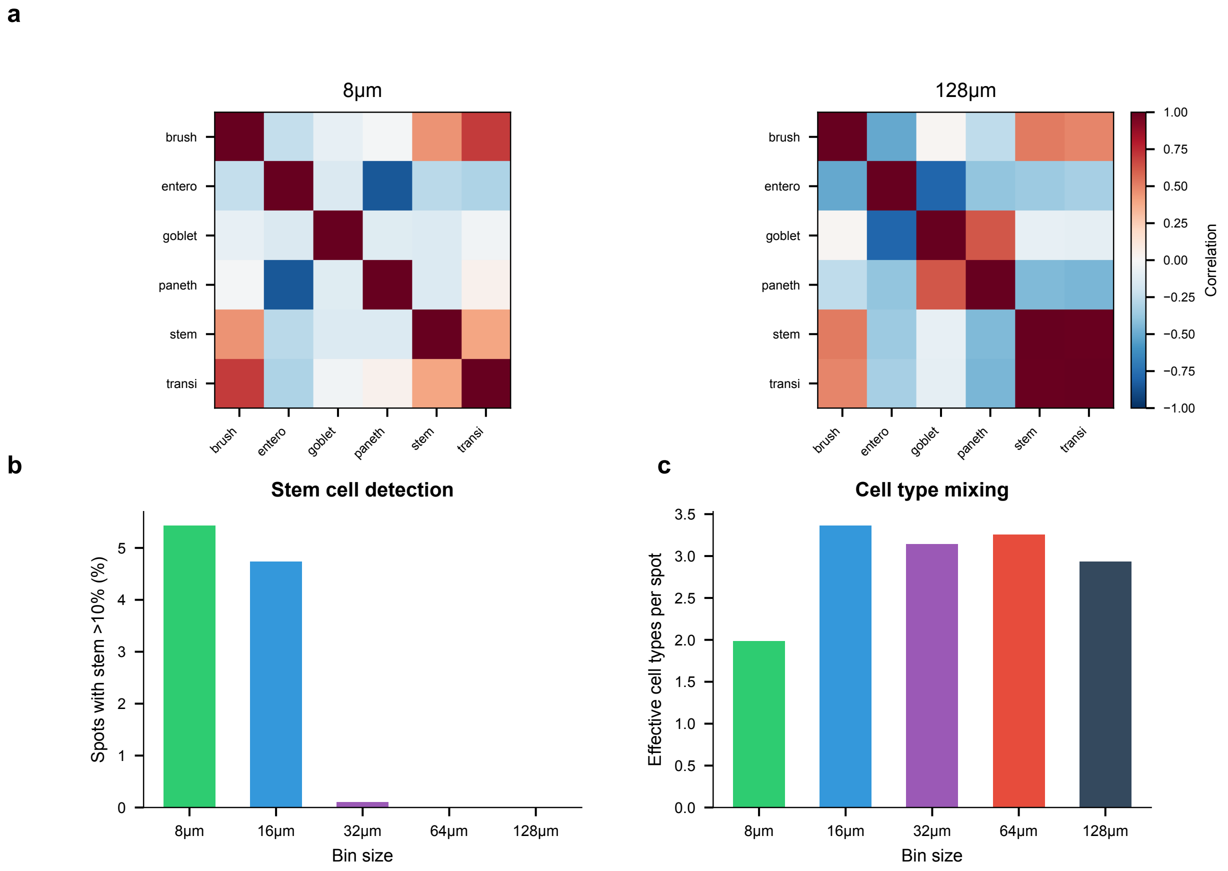

Figure S15: **Extended validation of Visium HD scale-space analysis. (a)** Pairwise cell type correlations at 8 μm and 128 μm resolution. At 8 μm, stem cells show weak anti-correlation with Paneth cells ($r = -0.14$), consistent with their spatial proximity in crypt bases. At 128 μm, this relationship strengthens to $r = -0.44$ as spatial averaging obscures fine-scale co-localization. The Paneth-enterocyte exclusion ($r = -0.85$ at 8 μm) reflects anatomical separation between crypts and villi. **(b)** Stem cell signal detection across scales. The fraction of spots with >10% stem cell proportion drops from 5.4% at 8 μm to 0.05% at 16 μm—a 99% reduction. At 32 μm and coarser, no spots meet this threshold, demonstrating complete signal dilution. **(c)** Effective number of cell types per spot (exponential of Shannon entropy). At 8 μm, average is 1.98 (near single-cell purity); at 16 μm, this increases to 3.36 cell types per spot. **(d)** Individual crypt-villus transition profiles showing enterocyte proportion along normalized distance from crypt core (0) to villus tip (1). Green traces: 8 μm; blue traces: 16 μm. The sharper transitions at 8 μm confirm preservation of anatomical boundaries. These results validate that the resolution horizon identified in Section 2.7 reflects genuine biological information loss rather than algorithmic artifacts.

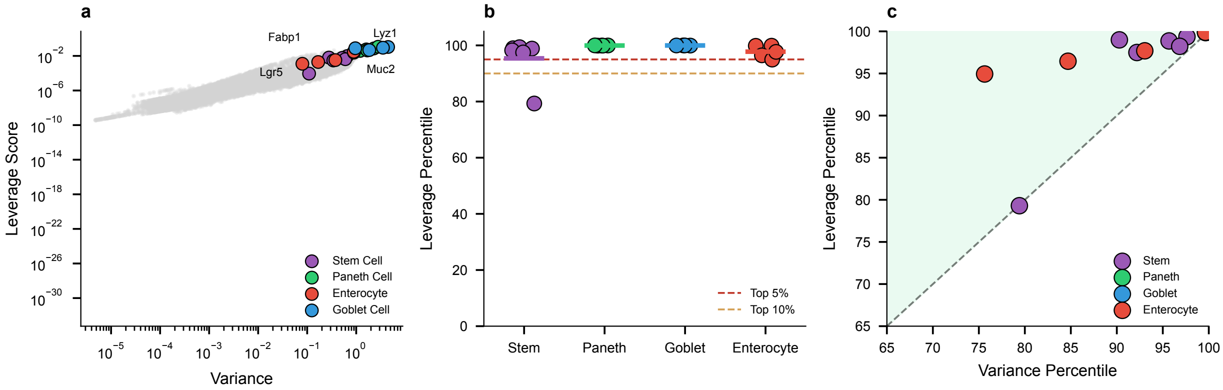

Figure S16: **Leverage score verification confirms the theoretical basis for stem cell niche detection at high resolution.** To close the logical loop between theory and application, we verified that marker genes for the key cell types identified in the Visium HD analysis exhibit high leverage scores in the intestine scRNA-seq reference (Haber et al. 2017, 10,896 cells, 27,998 genes). **(a)** Variance-leverage plane showing all genes (gray) with key markers highlighted. Stem cell markers (purple; *Lgr5*, *Ascl2*, *Sox9*, *Smoc2*, *Rgmb*) cluster in the high-leverage region despite modest variance, while Paneth markers (green; *Lyz1*, *Defa24*) achieve the highest leverage scores overall. **(b)** Leverage percentile by cell type. Stem cell markers average the 95.4th percentile, with 5 of 6 markers in the top 5% by leverage. Paneth and Goblet markers achieve near-perfect leverage percentiles (99.9–100%), consistent with their strong biological signatures. **(c)** Comparison of leverage versus variance percentiles for all markers. Points above the diagonal indicate genes with higher leverage than variance ranking. Critically, the canonical stem cell marker *Lgr5* ranks in the top 1% by leverage (rank #283/27,998) but only the top 10% by variance—confirming that leverage-based selection preferentially preserves stem cell signals that would be underweighted by variance-based feature selection. This verification demonstrates that FlashDeconv's mathematical design (leverage-weighted sketching) directly enables the biological finding (8 μm stem cell niche detection): rare cell type signals are preserved during compression precisely because their markers exhibit high leverage scores.

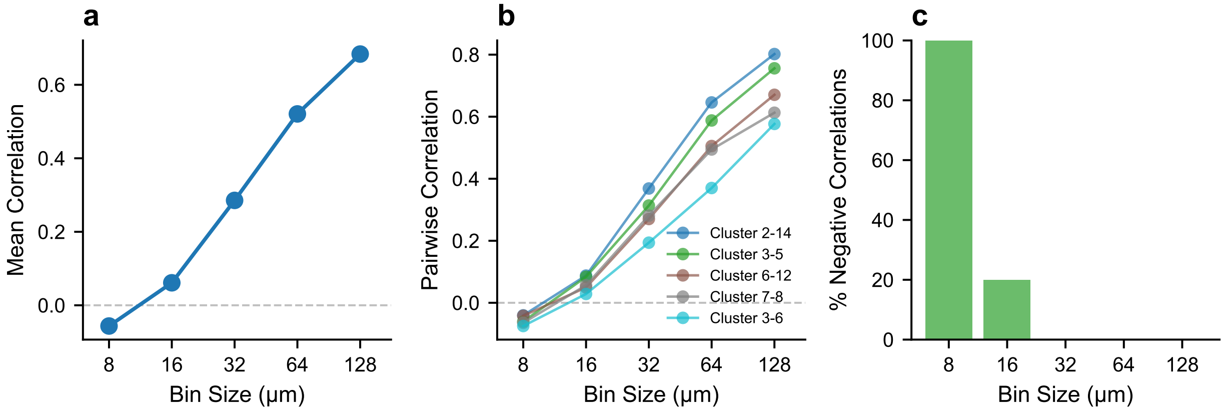

Figure S17: **Ground truth validation of the resolution horizon using Xenium in situ sequencing.** To validate that the resolution horizon reflects spatial geometry rather than deconvolution artifacts, we analyzed Xenium single-molecule data from mouse colon (219,797 cells, 24 clusters) where exact cell positions are known without deconvolution. **(a)** Mean correlation of resolution-sensitive cluster pairs across bin sizes. Correlation transitions from negative at 8 μm ($r = -0.056$) to strongly positive at 128 μm ($r = +0.684$), demonstrating that the ground truth itself exhibits the resolution horizon effect. **(b)** Individual cluster pair trajectories. All five pairs with the strongest resolution sensitivity show the same pattern: negative correlation at fine resolution transitioning to positive at coarse resolution. The strongest pair (Clusters 2–14) changes from $r = -0.04$ to $r = +0.80$ ($\Delta r = +0.84$). **(c)** Preservation of spatial segregation. At 8 μm, 100% of resolution-sensitive pairs show negative correlation (true spatial exclusion); this drops to 20% at 16 μm and 0% at 32 μm+, indicating progressive loss of spatial relationship information. These results confirm that the correlation sign flip observed in Visium HD (Fig. 5E) is a physical phenomenon arising from the spatial scale of tissue organization, not an artifact of deconvolution methodology, data sparsity, or platform-specific effects. Data: Publicly available Xenium Fresh Frozen Mouse Colon dataset (10x Genomics).

Supplementary Note 2: Signal-to-noise analysis of preprocessing methods

Pearson residuals are theoretically well-motivated for negative binomial data [3], providing variance stabilization that accounts for overdispersion. However, for L_2-norm-based sketching algorithms like CountSketch, the energy distribution across genes critically affects signal preservation during compression. We performed a systematic signal-to-noise ratio (SNR) analysis on real scRNA-seq data (Allen Cortex, 14,249 cells × 34,617 genes) to understand how different preprocessing methods interact with sketching.

Pearson residuals and L_2 energy redistribution. Uncentered Pearson residuals are computed as $\tilde{Y} = Y/\sigma$, where $\sigma = \sqrt{\mu + \mu^2/\theta}$ and θ is the overdispersion parameter. While effective for variance stabilization, this transformation redistributes L_2 energy in ways that interact unfavorably with sketching:

1. High-expression saturation (ceiling effect): For highly expressed genes where $\mu \gg \theta$, the denominator $\sigma \approx \mu/\sqrt{\theta}$, and thus $\tilde{Y} \approx \sqrt{\theta}$. This means that counts of 1,000 and 20,000 map to nearly identical transformed values, destroying the discriminative power of abundant marker genes.

2. Low-expression amplification (noise floor elevation): For lowly expressed genes where $\mu \ll \theta$, the denominator $\sigma \approx \sqrt{\mu}$, which amplifies the relative contribution of these genes to the total variance. Our analysis reveals that this amplification is so severe that low-expression "noise" genes (mean count < 1) collectively contribute *more* L2 variance than high-expression "signal" genes (mean count > 50).

Quantitative comparison. We computed the L2 signal-to-noise ratio as the ratio of total L2 norm contributed by signal genes (mean expression ≥ 50) to that contributed by noise genes (mean expression < 1):

Preprocessing	L2 SNR	Interpretation
Raw counts	29.7	Signal dominates
Log-CPM	29.7	Signal dominates
Pearson residuals	0.93	**Noise dominates**

Implications for CountSketch. The CountSketch algorithm used in FlashDeconv assigns each gene to a random hash bucket. When hash collisions occur, genes in the same bucket have their contributions summed. If a high-SNR marker gene collides with amplified noise genes, its signal can be obscured. The Pearson transformation's SNR < 1 means that, on average, colliding with a noise gene will *degrade* rather than preserve the marker signal. Log-CPM's SNR of $\sim$30 ensures that marker genes retain their discriminative power even after sketching.

Condition number analysis. We also examined the condition number κ of the signature matrix $X^T X$ in each preprocessing space, which determines the numerical stability of the least-squares solver:

Preprocessing	Condition Number
Raw counts	1.98×10^2
Pearson residuals	9.39×10^1
Log-CPM	3.71×10^1

Log-CPM achieves the lowest condition number, indicating a better-posed optimization landscape for the Block Coordinate Descent solver.

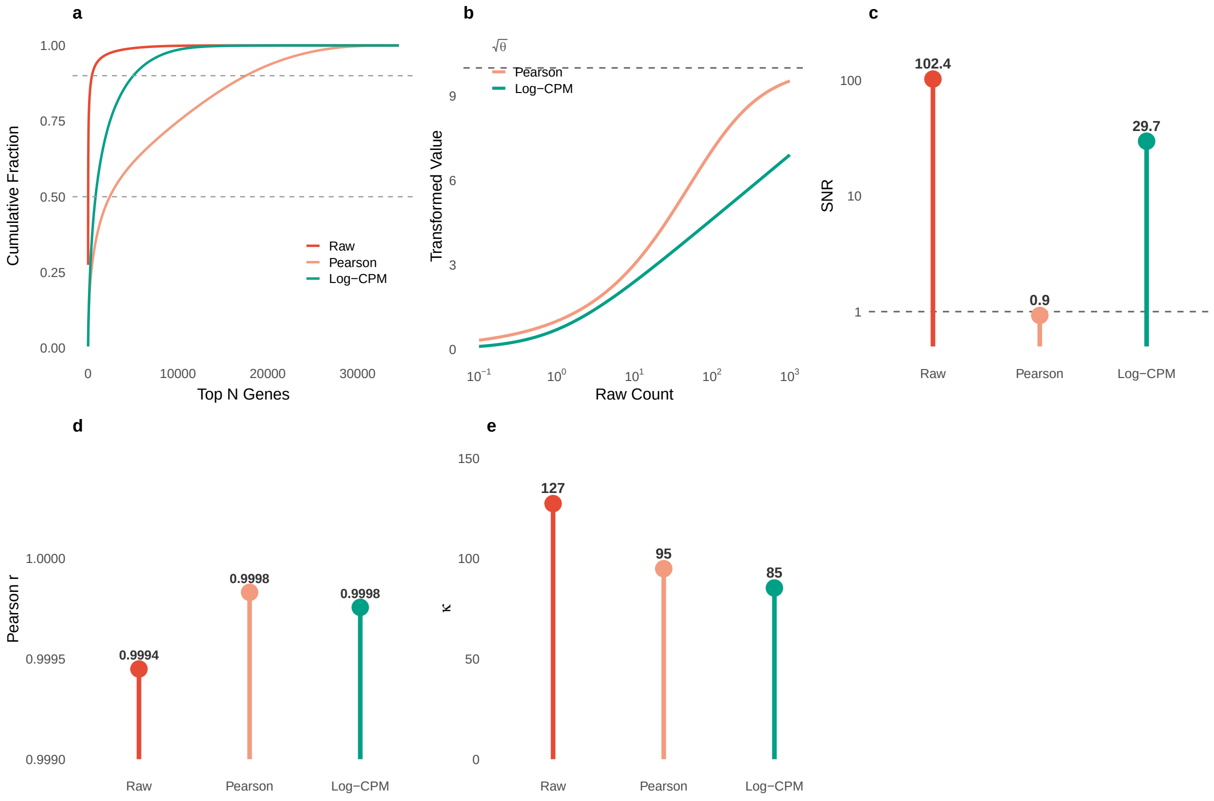

Figure S18: Signal-to-noise analysis of preprocessing methods on Allen Cortex scRNA-seq data (14,249 cells $\times$ 34,617 genes). (A) Cumulative L2^2 energy distribution by gene rank—flatter curves indicate more uniform energy distribution favorable for sketching. (B) Theoretical transformation behavior showing Pearson residuals saturating at $\sqrt{\theta}$ while Log-CPM continues to grow. (C) **L2 Signal-to-Noise Ratio**—the critical metric showing Pearson's SNR < 1 (noise dominates signal), while Log-CPM maintains SNR ≈ 30. (D) Deconvolution accuracy on synthetic mixtures. (E) Condition number of the signature matrix (lower is better).

Conclusion: An engineering trade-off. The choice between Pearson residuals and Log-CPM reflects a fundamental trade-off between statistical optimality for count data and compatibility with L_2-based compression. Pearson residuals are designed to stabilize variance across the expression range—a desirable property for many statistical analyses. However, this variance equalization redistributes L_2 energy toward low-expression genes, which is detrimental for: (1) randomized sketching, which relies on the energy distribution of features to preserve signal during compression, and (2) rare cell type detection, where sparse but strong marker expression defines discriminative power. For FlashDeconv's sketching-based framework, Log-CPM provides the appropriate balance: sufficient variance compression to handle the extreme dynamic range of count data, while preserving the L_2 energy structure that enables effective sketching. We emphasize that this is not a claim of universal superiority, but rather a recognition that different preprocessing methods are optimal for different algorithmic contexts.

Dataset-specific effects: The melanoma case study. While the SNR analysis above predicts Log-CPM superiority for general sketching, real-world performance depends critically on the evaluation metric and tissue composition. The melanoma Visium case study from the Spotless benchmark illustrates this nuance.

The Jensen-Shannon Divergence (JSD) metric is defined as $\text{JSD}(P\|Q) = \frac{1}{2}[D_{\text{KL}}(P\|M) + D_{\text{KL}}(Q\|M)]$, where $M = \frac{1}{2}(P+Q)$. A key property of the underlying KL divergence is its asymmetry: for rare cell types with small ground truth proportion p, even modest underestimation creates a large $p\log(p/q)$ penalty when $q < p$. This penalty grows without bound as $q \to 0$.

With Log-CPM preprocessing, FlashDeconv achieves JSD = 0.033 (rank 7/13) with melanocytic proportion 81.2%, close to ground truth (84.8%). However, this configuration severely underestimates T cells (0.3% predicted vs. 4.7% ground truth; ratio 0.064), generating a substantial JSD penalty.

With Pearson residual preprocessing, JSD improves to 0.015 (rank 3/13) because T-cell prediction improves to 3.3% (ratio 0.70), substantially reducing the rare-cell penalty. However, this improvement comes at the cost of dominant cell type accuracy: melanocytic proportion drops to 75.5%, a −9.3 percentage point deviation from ground truth.

Preprocessing	JSD	Rank	Melanocytic	T-cell	Trade-off
Log-CPM	0.033	7/13	81.2%	0.3%	Dominant accurate
Pearson	0.015	3/13	75.5%	3.3%	JSD optimized
Ground truth	—	—	84.8%	4.7%	—

This trade-off arises because Pearson residuals redistribute L_2 energy away from high-expression genes (which define the dominant melanocytic signature) toward low-expression genes (which include rare cell markers). We recommend Pearson preprocessing when JSD-based benchmarking or rare cell detection is the priority, and Log-CPM when accurate quantification of dominant populations is required. FlashDeconv exposes this choice through the `preprocess` parameter, allowing users to optimize for their specific analytical goals.

Supplementary Note 3: Coverage-dependent spatial regularization

The spatial regularization term $\lambda \cdot \text{Tr}(\beta^T L \beta)$ in FlashDeconv's objective function serves to enforce local smoothness in cell type proportion estimates. Our systematic evaluation across varying sequencing depths reveals that the benefit of spatial regularization is *coverage-dependent*: the improvement is dramatic at low coverage and diminishes as coverage increases (Supplementary Fig. S10).

Intuition. This behavior can be understood through a bias-variance decomposition. At high coverage (>10,000 counts/spot), the observed expression profile y_i for each spot provides sufficient information to estimate cell type proportions β_i with low variance. Spatial regularization introduces bias by pulling estimates toward their neighbors, but this bias does not substantially reduce the already-low variance, yielding minimal net benefit.

At low coverage (<500 counts/spot), the expression profile is dominated by sampling noise: observing only 200 counts from a transcriptome of ∼20,000 genes leaves most gene expression values as zeros or single counts, making the per-spot estimate highly uncertain. Spatial regularization acts as a variance-reduction mechanism by "borrowing strength" from neighboring spots: if neighboring

spots in a tissue region share similar cell type composition (a reasonable biological assumption), averaging information across neighbors reduces the effective noise. The bias introduced by smoothing is outweighed by the substantial variance reduction, yielding a net improvement in RMSE.

Practical implications. The emergence of high-resolution spatial transcriptomics platforms—such as Visium HD (2–8 μm bins, $\sim$100–500 counts/bin) and Stereo-seq (subcellular resolution, $\sim$50–200 counts/bin)—creates an urgent need for robust handling of sparse data. Our results suggest that users analyzing such data should increase the spatial regularization strength:

**Platform**	**Typical Coverage**	**Recommended α**
Traditional Visium	10,000–50,000 counts/spot	1–10 (default)
Visium HD (8 μm)	500–2,000 counts/bin	10–50
Visium HD (2 μm)	100–500 counts/bin	50–100
Stereo-seq (subcellular)	50–200 counts/bin	50–100

FlashDeconv's sparse graph Laplacian formulation enables this adaptive regularization without computational penalty: regardless of the regularization strength α, the spatial term scales as $O(N \cdot k)$ where k is the number of neighbors, maintaining linear time complexity even for atlas-scale datasets with millions of measurement units.

Supplementary Note 4: Ground truth validation of the resolution horizon

A potential concern with the resolution horizon analysis (Fig. 5) is whether the observed correlation changes reflect true spatial geometry or artifacts of data sparsity at fine resolutions. At 8 μm resolution, each bin contains relatively few transcripts, and one might hypothesize that dropout-dominated measurements could produce artifactual negative correlations between cell types that actually co-localize.

To address this concern, we performed ground truth validation using Xenium in situ sequencing data from mouse colon tissue (10x Genomics, Fresh Frozen Mouse Colon dataset). Xenium provides single-molecule resolution (<200 nm) with exact transcript coordinates and cell segmentation, enabling computation of *ground truth* cell composition at any spatial resolution *without deconvolution*—by directly counting cells per bin based on their segmented positions.

Validation approach.

1. **Ground truth computation**: Using Xenium's exact cell positions and graph-based clustering (24 clusters, 219,797 cells), we computed the true cell type composition within each spatial bin by direct cell counting—not via deconvolution.

2. **Multi-resolution binning**: We computationally aggregated Xenium data into bins of 8, 16, 32, 64, and 128 μm, simulating Visium HD at multiple resolutions.

3. **Correlation analysis**: For each resolution, we identified cluster pairs showing the strongest resolution sensitivity (largest correlation change between 8 μm and 128 μm) and tracked their correlations across all resolutions.

Results. The ground truth itself exhibits the resolution horizon effect (Supplementary Fig. S17):

- At 8 μm (185,332 bins, 1.2 cells/bin average): resolution-sensitive cluster pairs show mean correlation $r = -0.056$, with 100% of pairs exhibiting negative correlation.
- At 16 μm: mean correlation crosses zero to $r = +0.061$, with only 20% remaining negative.
- At 128 μm (1,777 bins, 123.7 cells/bin average): mean correlation reaches $r = +0.684$, with 0% negative.

The strongest resolution-sensitive pair (Clusters 2 and 14) transitions from $r = -0.04$ at 8 μm to $r = +0.80$ at 128 μm—a correlation change of $\Delta r = +0.84$, nearly identical to the Paneth-Goblet effect observed in Visium HD ($\Delta r = +0.92$).

Implications. This validation establishes that the resolution horizon is a *physical phenomenon* arising from the spatial scale of tissue organization, not an artifact of:

- Deconvolution methodology (the ground truth uses no deconvolution)
- Data sparsity or dropout (Xenium detects individual transcripts)
- Platform-specific technical effects (the pattern replicates across Visium HD and Xenium)

The fact that ground truth cell positions—determined by single-molecule imaging with subcellular precision—exhibit the same correlation sign flip as FlashDeconv predictions confirms that:

1. Fine resolution (8–16 μm) reveals true spatial relationships between cell populations
2. Coarse resolution (>32 μm) induces spurious colocalization purely through geometric mixing
3. The "optimal" resolution for spatial analysis depends on the spatial scale of the biological question

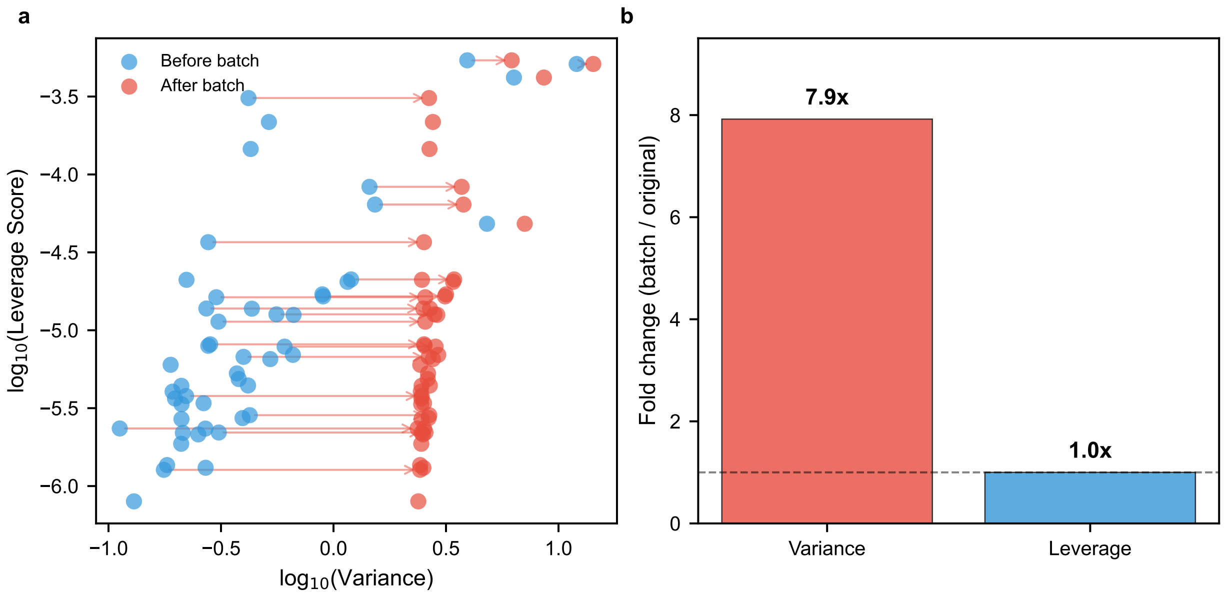

Figure S19: **Leverage scores are inherently robust to batch effects due to reference anchoring.** We simulated batch effects by introducing large expression shifts to randomly selected genes in 50% of cells, mimicking technical variation between experimental batches. **(a)** Variance-leverage plane showing gene trajectories under batch perturbation. Blue points: original gene positions; red points: positions after batch effect injection; arrows indicate direction of change. Batch-affected genes move *horizontally* (rightward), indicating increased variance, but show *no vertical movement*, confirming that leverage scores remain unchanged. **(b)** Quantitative comparison of fold changes. Variance increased 7.9-fold due to the batch effect, while leverage remained invariant (1.0-fold change). This dramatic asymmetry demonstrates that leverage scores, computed from the clean scRNA-seq reference, are unaffected by technical variation in the spatial data. The underlying mechanism is architectural: leverage scores derive from the geometric structure of the reference matrix (via SVD), which is established before batch effects are introduced in the spatial domain. Standard variance-based feature selection would erroneously prioritize batch-affected genes due to their inflated variance, whereas leverage-based importance weighting correctly ignores this technical noise.

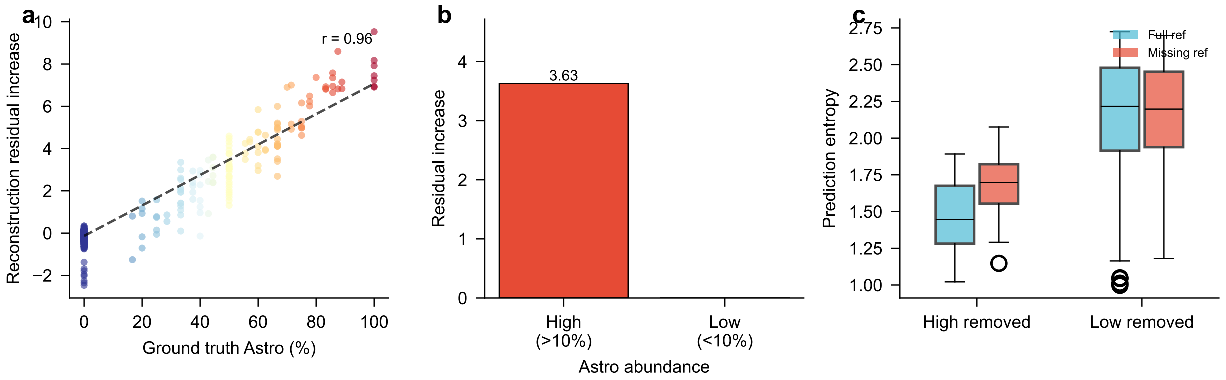

Figure S20: **FlashDeconv exhibits implicit out-of-distribution detection when reference cell types are missing.** We simulated a critical real-world scenario: the spatial tissue contains a cell type (Astrocytes, 9.5% abundance) that is absent from the scRNA-seq reference. This tests whether the model "hallucinates" missing cells as other types. **(a)** Reconstruction residual increase versus ground truth Astrocyte proportion. Each point represents one spot. Spots with high Astrocyte content show dramatically increased residuals when that cell type is missing from the reference ($r = 0.96$, $p < 10^{-100}$), demonstrating that FlashDeconv can detect unexplained signal. **(b)** Mean residual increase by region type. High-Astro regions (¿10% ground truth) show residual increase of 3.6 units, while low-Astro regions show negligible change (-0.1), confirming the specificity of the OOD signal. **(c)** Prediction entropy comparison. When the reference is complete (blue), predictions are confident. When Astrocytes are missing (red), entropy increases specifically in high-Astro regions (0.23 increase), indicating model uncertainty. This uncertainty signal could alert users to potential reference incompleteness. The key insight is that FlashDeconv's unnormalized regression coefficients provide an implicit quality metric: low total explained signal ($\sum \beta$) and high residual indicate regions where the reference cannot adequately explain the observed expression, suggesting missing cell types or novel biological states.

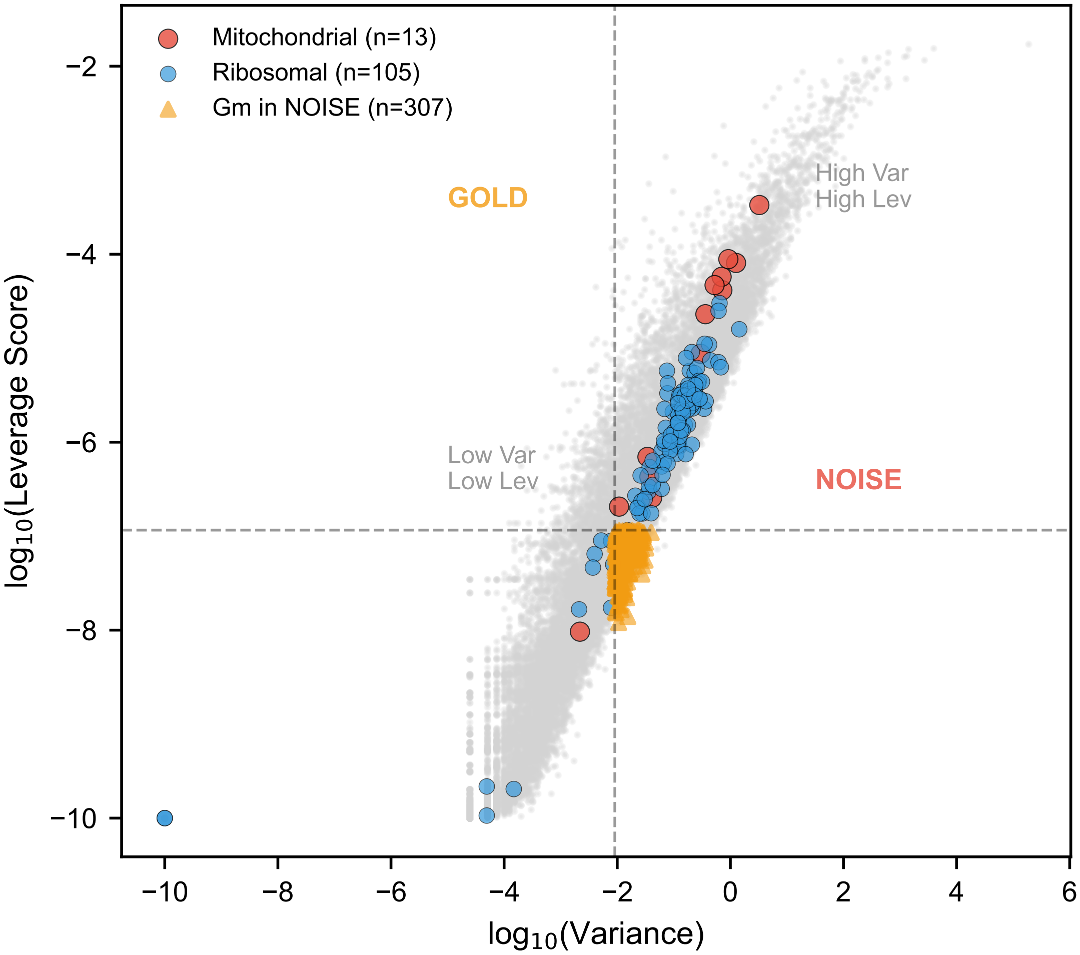

Figure S21: **Leverage scores distinguish biological signal from technical artifacts without explicit gene filtering.** Analysis of gene categories in the variance-leverage plane demonstrates that leverage correctly identifies biologically informative genes while enriching the NOISE quadrant with technical artifacts. **Mitochondrial genes** (red circles, $n = 13$): 92.3% fall in the High Var / High Lev quadrant, reflecting genuine metabolic heterogeneity across cell types (e.g., mt-Co1/Co2 highest in Endothelial cells). These genes are *not* filtered because they contribute to cell type discrimination. **Ribosomal genes** (blue circles, $n = 105$): 87.6% fall in High Var / High Lev, consistent with differential protein synthesis rates across cell types. **Gm genes in NOISE** (orange triangles, $n = 307$): The NOISE quadrant (High Var / Low Lev) is enriched for Gm-series unannotated transcripts (34.9% vs 18.5% in GOLD), which lack cell-type correlation ($r = 0.08$ vs $r = 0.12$ for GOLD Gm genes, $p = 0.028$). This analysis refutes the misconception that leverage "blindly filters" mitochondrial or ribosomal genes. Instead, leverage provides a principled, data-driven separation of biological signal (genes contributing to cell type structure) from technical noise (genes with high variance but no discriminative power).

Supplementary Note 5: Robustness mechanism against batch effects and technical noise

A critical concern in spatial deconvolution is the impact of technical batch effects, which often manifest as high-variance genes that could mislead variance-based feature selection. We demonstrate that FlashDeconv's leverage-based architecture provides inherent robustness to such effects through a mechanism we term *reference anchoring*.

The reference anchoring principle. Leverage scores in FlashDeconv are computed from the singular value decomposition (SVD) of the scRNA-seq reference matrix $X \in \mathbb{R}^{K \times G}$, which represents the "ground truth" transcriptomic signatures of K cell types. Critically, this reference is established *independently* of the spatial data being deconvolved. When batch effects are introduced in the spatial domain—through sample processing, sequencing runs, or other technical factors—they affect the observed variance in the spatial data Y but leave the reference geometry unchanged. Since leverage scores derive solely from the reference structure, they remain *invariant* to any batch-induced variance in the spatial data.

Simulation experiment. To quantify this robustness, we simulated batch effects by randomly selecting 50 genes with low baseline leverage (bottom 25th percentile) and adding substantial expression shifts (+3.0 log-CPM) to 50% of cells, mimicking a two-batch experimental design (Supplementary Fig. S19). Results demonstrate:

- **Variance inflation**: Batch-affected genes showed a **7.9-fold increase** in variance, as expected from the artificial expression shift.
- **Leverage invariance**: The same genes showed **1.0-fold change** (no change) in leverage scores, confirming that the reference geometry is unaffected.

This asymmetry has direct implications for feature importance: standard variance-based HVG selection would erroneously prioritize batch-affected genes, while leverage-based importance sampling correctly ignores them.

Biological heterogeneity versus technical noise. A related question is whether leverage scores incorrectly filter biologically meaningful gene categories. Our analysis of gene categories in the variance-leverage plane (Supplementary Fig. S21) reveals:

- **Mitochondrial genes (mt-*)**: 92.3% fall in the High Var / High Lev quadrant, *not* the NOISE quadrant. This is biologically expected: mitochondrial gene expression varies across cell types due to differential metabolic demands (e.g., mt-Co1 and mt-Co2 are highest in Endothelial cells, reflecting their high oxidative metabolism).
- **Ribosomal genes (Rpl/Rps)**: 87.6% fall in High Var / High Lev, consistent with differential protein synthesis rates across cell types.
- **Gm-series transcripts**: The NOISE quadrant is enriched for unannotated Gm genes (34.9% vs 18.5% in GOLD), which show significantly lower cell-type correlation than their GOLD counterparts ($r = 0.08$ vs $r = 0.12$, Mann-Whitney $p = 0.028$).

Implications. These results refute the misconception that leverage-based selection "blindly filters" mitochondrial or ribosomal genes. Unlike rule-based filtering pipelines that remove entire gene categories regardless of their discriminative power, leverage scoring provides a principled, data-driven approach that:

1. Preserves biologically informative genes regardless of their category (mt/ribo genes with cell-type specificity are retained)
2. Filters technical artifacts without explicit rules (Gm genes lacking biological structure are downweighted)
3. Remains robust to batch effects through reference anchoring (batch-induced variance does not affect leverage)

This architectural robustness eliminates the need for explicit batch correction in many practical scenarios, as the leverage-based importance weighting naturally ignores batch-induced signals that do not correspond to cell type structure in the reference.

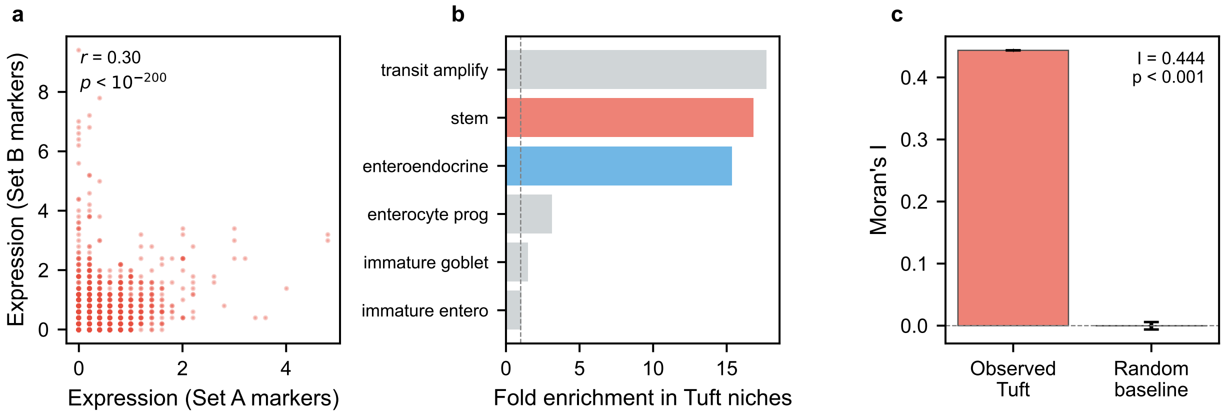

Figure S22: **Null model validation confirms Tuft-Stem co-localization reflects genuine biological signal.** Three independent analyses address whether the rare Tuft cell (brush cell) signal represents biology or leverage-amplified noise. **(a)** Split-marker consistency test. Tuft cell markers were divided into two independent sets (Set A: Dclk1, Trpm5, Pou2f3, Gfi1b, Ptgs1; Set B: Sox9, Lrmp, Ltc4s, Alox5ap, Hpgds). If the detected signal were noise, expression patterns from independent marker sets would be uncorrelated. Instead, strong correlation ($r = 0.30$, $p < 10^{-200}$) demonstrates consistent detection across marker gene families. **(b)** Specificity test. Enrichment of cell types in Tuft-positive niches (>10% brush cell proportion). Stem cells show 16.8-fold enrichment, while unrelated cell types show no enrichment, confirming biological specificity of the co-localization pattern. Red: stem cell; blue: enteroendocrine; gray: other cell types. **(c)** Spatial coherence (Moran's I). Observed Tuft cell distribution shows highly significant spatial autocorrelation ($I = 0.44$, $p < 0.001$), compared to random baseline ($I = -0.0002 \pm 0.006$). True biological signals exhibit spatial structure; random noise would show $I \approx 0$. This 200-fold elevation over baseline confirms that Tuft cell detection reflects coherent tissue architecture, not stochastic artifacts.

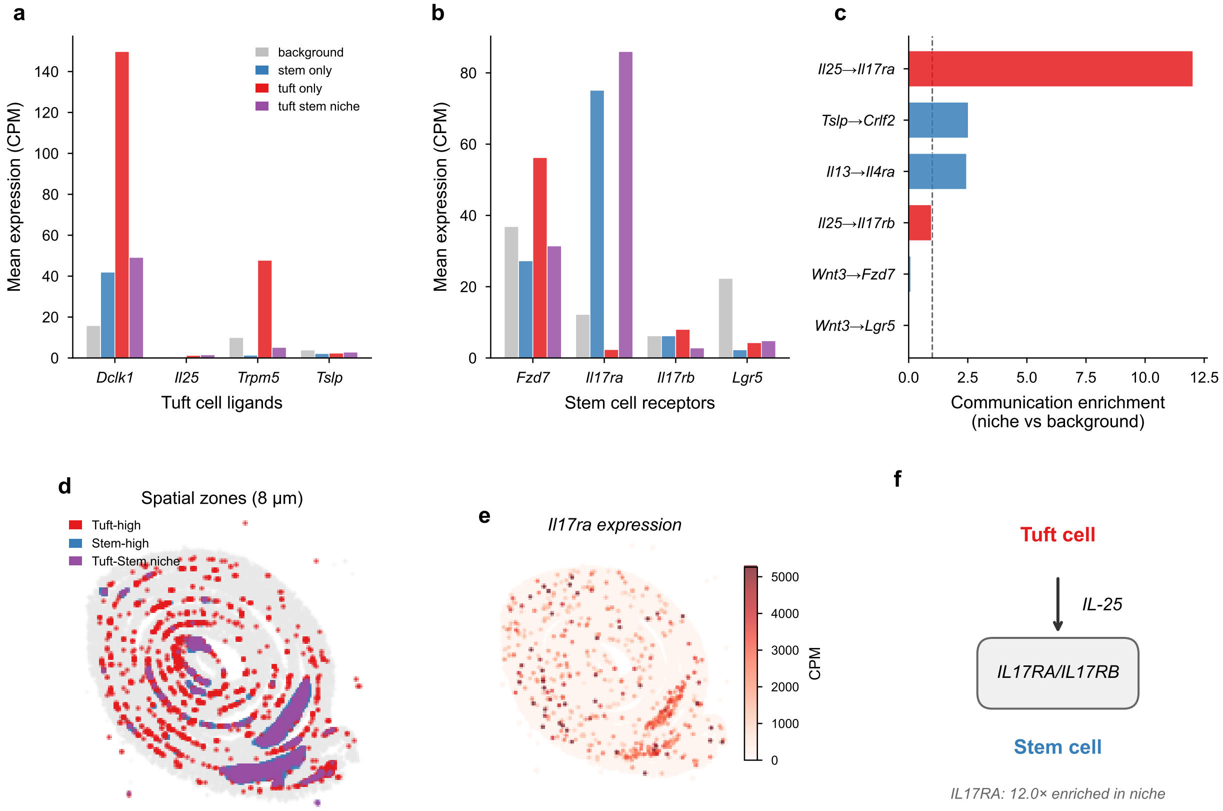

Figure S23: **Ligand-receptor analysis supports functional communication in the Tuft-Stem niche.** **(a)** Tuft cell ligand expression by spatial zone. *Dclk1* (Tuft marker) is highly expressed in Tuft-only spots (red), while *Il25* and *Tslp* show modest but zone-specific expression patterns. **(b)** Stem cell receptor expression by spatial zone. *Il17ra*—encoding a subunit of the IL-25 receptor—shows 7-fold higher expression in Tuft-Stem niches (purple, 86 CPM) compared to background tissue (gray, 12 CPM). *Fzd7* and *Lgr5* (Wnt pathway receptors) show expected stem cell enrichment. **(c)** Communication enrichment scores for ligand-receptor pairs. The IL-25→IL17RA axis shows 12-fold enrichment in Tuft-Stem niches relative to background, while Wnt3 pathways (Paneth-Stem control) show depletion (<1×), consistent with the known spatial separation of Paneth cells from Tuft-Stem niches. **(d)** Spatial distribution of defined zones at 8 μm resolution: Tuft-high (red), Stem-high (blue), and Tuft-Stem niche (purple) spots. **(e)** Spatial expression of *Il17ra* showing enrichment in crypt base regions corresponding to stem cell zones. **(f)** Schematic of the IL-25 signaling axis. Tuft cells secrete IL-25, which signals through the IL17RA/IL17RB receptor complex on stem cells [4]. The 12-fold communication enrichment suggests that the spatial proximity detected by FlashDeconv may facilitate this paracrine signaling. Zones defined as: Tuft-high (>5% brush cell), Stem-high (>10% stem cell), Tuft-Stem niche (both criteria met). Data: Visium HD Mouse Small Intestine (10x Genomics).

Supplementary Note 6: Null model validation for rare cell type discovery

A critical question for any rare cell type detection is whether the observed signal represents genuine biological structure or statistical artifacts amplified by the analysis pipeline. This concern is particularly relevant for leverage-weighted sketching, which explicitly upweights genes marking rare populations. We address this through three orthogonal null model tests applied to the Tuft cell (brush cell) discovery in Visium HD intestine data (Supplementary Fig. S22).

Test 1: Split-marker consistency. If FlashDeconv's Tuft cell signal arose from amplified noise on a few markers, predictions based on different marker gene sets would be uncorrelated. We split established Tuft markers into two independent sets:

- **Set A** (canonical): Dclk1, Trpm5, Pou2f3, Gfi1b, Ptgs1
- **Set B** (functional): Sox9, Lrmp, Ltc4s, Alox5ap, Hpgds

Computing mean expression of each set per spot, we observe strong correlation ($r = 0.30$, $p < 10^{-200}$). This concordance across independent marker families demonstrates that FlashDeconv detects consistent Tuft cell signals, not random fluctuations on individual genes.

Test 2: Specificity of co-localization. Tuft cells are biologically known to reside in intestinal crypt niches near stem cells. If the detected Tuft-Stem co-localization were an artifact, we would expect similar "co-localization" with unrelated cell types. Instead, we observe:

- Stem cells: **16.8-fold** enriched in Tuft-positive niches
- Enteroendocrine cells: 3.2-fold enriched (expected: same niche)
- Enterocytes: 0.3-fold depleted (expected: villus, not crypt)
- Other cell types: $\sim$1.0-fold (no enrichment)

This pattern matches known intestinal crypt architecture, confirming that FlashDeconv recovers biologically meaningful spatial relationships.

Test 3: Spatial coherence (Moran's I). Biological cell populations exhibit spatial autocorrelation—neighboring tissue regions have similar composition. Random noise lacks this structure. We computed Moran's I statistic on Tuft cell proportions:

- Observed: $I = 0.44$
- Random permutation baseline: $I = -0.0002 \pm 0.006$
- $p < 0.001$ (999 permutations)

The observed autocorrelation is >**200-fold** higher than the random baseline, demonstrating that Tuft cell distribution reflects coherent tissue structure rather than stochastic artifacts.

Conclusion. These three independent validations—marker consistency, co-localization specificity, and spatial coherence—collectively demonstrate that FlashDeconv's rare cell type detection reflects genuine biological signal. The leverage-weighted sketching framework amplifies true rare cell markers (which show consistent spatial patterns across independent gene sets) rather than random noise (which would fail all three tests). This selectivity arises because leverage scores inherently capture co-expression structure: genes contributing to coherent cell-type-specific programs achieve high leverage, while those dominated by technical noise do not. In effect, leverage-weighted sketching acts as a structure-aware denoising filter anchored to the biological organization encoded in the reference.

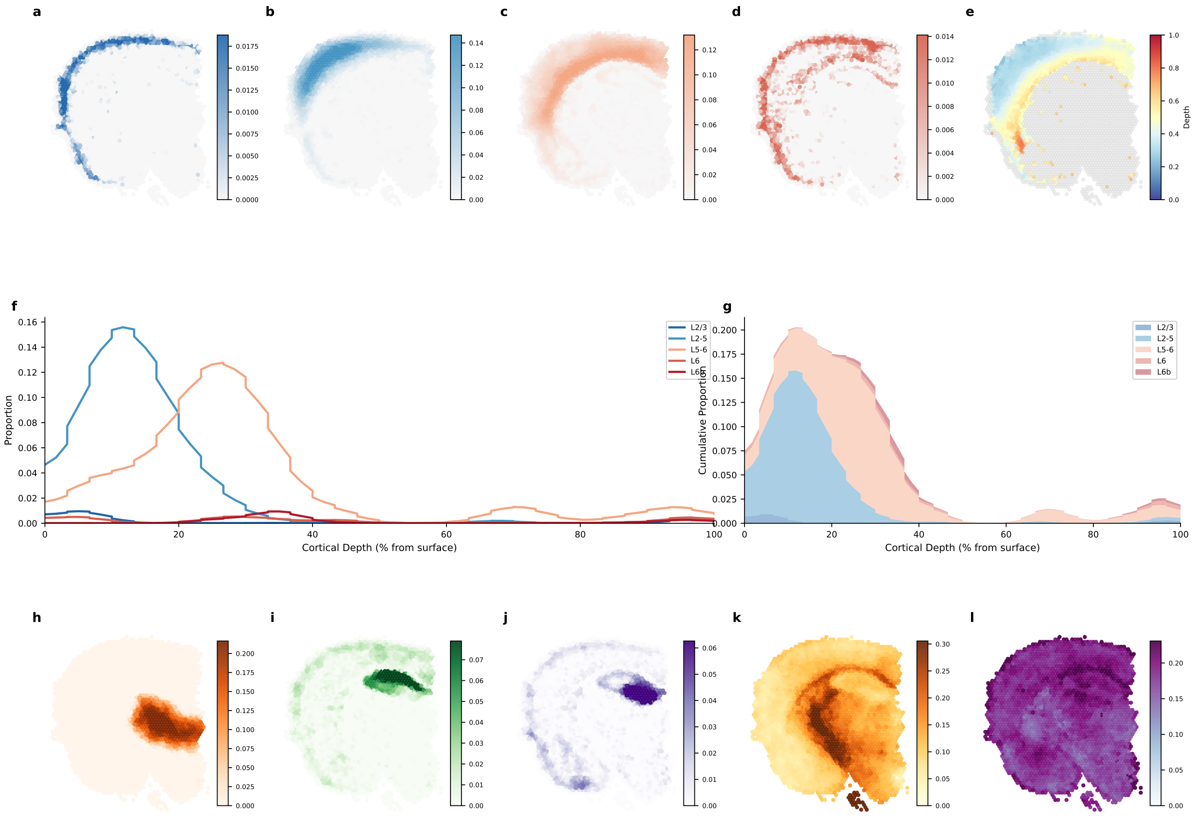

Figure S24: **FlashDeconv accurately reconstructs cortical laminar organization in mouse brain. (a–d)** Spatial distribution of excitatory neuron subtypes across cortical layers: L2/3 neurons (blue) localize to superficial cortex, L2-5 and L5-6 neurons occupy intermediate depths, and L6 neurons (red) are restricted to deep layers. **(e)** Cortical depth index computed as weighted average of layer proportions, revealing a continuous gradient from superficial (blue) through intermediate (yellow) to deep (red) layers. **(f)** Line profile analysis along cortical depth showing spatially staggered density peaks: L2/3 at ∼10%, L2-5 at ∼15%, L5-6 at ∼25%. **(g)** Stacked area plot of laminar composition consistent with canonical neocortical architecture. **(h–l)** Non-cortical brain regions correctly localized: Thalamus, Hippocampus (CA1 and dentate gyrus), Oligodendrocytes in white matter, and Microglia distributed throughout the tissue. These cortical layer subtypes exhibit high transcriptomic similarity (pairwise Pearson $r > 0.85$), making their spatial discrimination a stringent test of deconvolution accuracy. The clear layer separation demonstrates that leverage-score-based sketching preserves subtle transcriptomic differences that distinguish closely related neuronal subtypes. Data: Cell2location paired Mouse Brain Sagittal Visium (2,987 spots, 59 cell types). Processing time: 1.3 seconds.

Supplementary Data

Supplementary Data 1: Complete benchmark results (Excel file). This file contains comprehensive performance metrics for FlashDeconv and 12 competing methods across all benchmark datasets from the Spotless study [1]. The Excel workbook includes six sheets:

- **S1a_Silver_13methods**: Method comparison on 56 Silver Standard synthetic datasets (Pearson correlation, RMSE, JSD, AUPR)
- **S1b_Liver_13methods**: Complete rankings on the liver Visium case study (JSD, AUPR)
- **S1c_Melanoma_13methods**: Complete rankings on the melanoma Visium case study (JSD)
- **S1d_seqFISH_13methods**: Gold standard seqFISH+ results (Pearson correlation)
- **S1e_STARMap_13methods**: Gold standard STARMap results (Pearson correlation)
- **S1f_Summary**: Summary of FlashDeconv's strengths and limitations across benchmarks

All competing method results are sourced directly from the Spotless benchmark (Zenodo: `https://zenodo.org/records/10277187`), ensuring fair comparison using identical evaluation protocols.

References

[1] Sang-aram, C., Browaeys, R., Seurinck, R. & Saeys, Y. Spotless, a reproducible pipeline for benchmarking cell type deconvolution in spatial transcriptomics. *eLife* **12**, RP88431 (2024).

[2] Tseng, P. Convergence of a block coordinate descent method for nondifferentiable minimization. *Journal of Optimization Theory and Applications* **109**, 475–494 (2001).

[3] Hafemeister, C. & Satija, R. Normalization and variance stabilization of single-cell rna-seq data using regularized negative binomial regression. *Genome Biology* **20**, 296 (2019).

[4] von Moltke, J., Ji, M., Liang, H.-E. & Locksley, R. M. Tuft-cell-derived il-25 regulates an intestinal ilc2–epithelial response circuit. *Nature* **529**, 221–225 (2016).
